# Type I pyridoxal 5′-phosphate dependent enzymatic domains embedded within multimodular nonribosomal peptide synthetase and polyketide synthase assembly lines

**DOI:** 10.1186/1472-6807-13-26

**Published:** 2013-10-23

**Authors:** Teresa Milano, Alessandro Paiardini, Ingeborg Grgurina, Stefano Pascarella

**Affiliations:** 1 Dipartimento di Scienze Biochimiche “A. Rossi Fanelli”, Sapienza - Università di Roma, Roma 00185, Italy

**Keywords:** Pyridoxal 5′-phosphate, Fold type I, Nonribosomal peptide synthetases, Polyketide synthases, Tailoring domains, Hidden Markov models, Homology modeling, Protein-protein interaction, Docking

## Abstract

**Background:**

Pyridoxal 5′-phosphate (PLP)-dependent enzymes of fold type I, the most studied structural class of the PLP-dependent enzyme superfamily, are known to exist as stand-alone homodimers or homotetramers. These enzymes have been found also embedded in multimodular and multidomain assembly lines involved in the biosynthesis of polyketides (PKS) and nonribosomal peptides (NRPS). The aim of this work is to provide a proteome-wide view of the distribution and characteristics of type I domains covalently integrated in these assemblies in prokaryotes.

**Results:**

An ad-hoc Hidden Markov profile was calculated using a sequence alignment derived from a multiple structural superposition of distantly related PLP-enzymes of fold type I. The profile was utilized to scan the sequence databank and to collect the proteins containing at least one type I domain linked to a component of an assembly line in bacterial genomes. The domains adjacent to a carrier protein were further investigated. Phylogenetic analysis suggested the presence of four PLP-dependent families: Aminotran_3, Beta_elim_lyase and Pyridoxal_deC, occurring mainly within mixed NRPS/PKS clusters, and Aminotran_1_2 found mainly in PKS clusters. Sequence similarity to the reference PLP enzymes with solved structures ranged from 24 to 42% identity. Homology models were built for each representative type I domain and molecular docking simulations with putative substrates were carried out. Prediction of the protein-protein interaction sites evidenced that the surface regions of the type I domains embedded within multienzyme assemblies were different from those of the self-standing enzymes; these structural features appear to be required for productive interactions with the adjacent domains in a multidomain context.

**Conclusions:**

This work provides a systematic view of the occurrence of type I domain within NRPS and PKS assembly lines and it predicts their structural characteristics using computational methods. Comparison with the corresponding stand-alone enzymes highlighted the common and different traits related to various aspects of their structure-function relationship. Therefore, the results of this work, on one hand contribute to the understanding of the functional and structural diversity of the PLP-dependent type I enzymes and, on the other, pave the way to further studies aimed at their applications in combinatorial biosynthesis.

## Background

Pyridoxal 5′-phosphate (PLP), a derivative of Vitamin B_6,_ is one of the most versatile organic cofactors in biology. In fact, PLP-dependent enzymes form a vast and complex group of proteins present in organisms belonging to all levels of the tree of life [1] and participate in a variety of reactions (Additional file 1: Scheme 1). In humans, for example, besides the classical role in transamination, many of these enzymes take part in the metabolism of neurotransmitters such as dopamine, serotonin, glycine, epinephrine, norepinephrine, d-serine, l-glutamate, γ-amino butyric acid and histamine [2]. PLP-dependent enzymes have been classified in at least five evolutionarily unrelated families, characterized by specific three-dimensional folds [1,3]. Many studies have been devoted to the elaboration of a rigorous classification of PLP enzymes, with the aim to identify their common structural features and to understand how different protein scaffolds can support similar substrate binding in the active sites [4]. Among the different structural classes, the so-called fold type I [5] is the most populated in nature and the best characterized one. The subunit architecture of the fold includes one large and one small domain. The large domain contains a seven stranded β-sheet interacting with *α*-helices. The small domain at the C-terminal part of the chain folds as a three- or four-stranded β-sheet partly covered with helices. PLP-enzymes belonging to the fold type I, are known to exist as stand-alone proteins either homodimers or homotetramers. The active site is located in a crevice between the two domains at the subunit interface. The archetypal protein of this class is aspartate aminotransferase [6] which was the first PLP-dependent enzyme to be purified and crystallized. Since then, intense research work has been carried out to elucidate the details of its structural and functional properties.

Scrutiny of sequence data produced by genomic projects showed that fold type I domains can be found in multidomain frameworks, in prokaryotic systems, such as the transcriptional regulator MocR [7,8] and also in multienzyme systems, polyketide synthases (PKS) and nonribosomal peptide synthetases (NRPS), involved in the biosynthesis of polyketides (PK), nonribosomal peptides (NRP) and hybrid PK/NRP secondary metabolites. A vast array of bioactive metabolites belonging to these classes includes also several important medicinal agents and biotechnologically relevant compounds [9,10]. The canonical biosynthetic mechanisms of the two classes of structurally different secondary metabolites, polyketides (PK) and nonribosomal peptides (NRP), share several common features. Both NRPS and PKS type I systems require the participation of multienzyme complexes acting as assembly lines for the construction of polyketide or peptide chains by a sequence of condensation steps. In both systems each elongation step is catalyzed by a module containing the catalytic domains required for the insertion of a monomer into the growing chain. The first step in the biosynthetic pathway, is the ATP-dependent adenylation of the amino acid catalyzed by an adenylation domain (A) in the NRPS systems and the transfer of the acyl-group from an acyl-CoA onto the acyltransferase unit (AT) in the PKS systems. The monomer is then transferred to a carrier protein (CP) post-translationally primed with a phosphopantetheine arm, called thiolation domain (T) or peptidyl carrier protein (PCP) in NRPS and acyl carrier protein (ACP) in PKS multienzyme assemblies, respectively. In both systems, the carrier proteins mediate the transport of intermediates, linked by a thioester bond to the phosphopantetheine arm, along the assembly line. In NRPS systems, the key elongation reaction is the peptide bond formation by a nucleophilic attack of the α-amino group of the amino acid tethered to the downstream thiolation domain on the thioester bond of the intermediate tethered to the upstream peptidyl carrier protein, catalyzed by a condensation domain (C). In PKS assembly lines the elongation relies on the ketosynthase (KS) catalyzed carbon-carbon bond formation, by a Claisen condensation mechanism between the upstream acyl thioester and the downstream carbanionic acyl acceptor resulting from decarboxylation of malonyl- or methylmalonyl-ACP. The release of the product in both systems is usually catalyzed by a thioesterase (TE) domain located in the termination module and in most cases involves a macrocyclization; however different mechanisms have also been reported [9,10]. In addition to these two distinct biosynthetic mechanisms, there is a large number of mixed clusters involved in the production of structurally complex compounds where both polyketide and peptide moieties can be recognized. The modular architecture and functional versatility makes possible the switching between NRPS and PKS assembly lines.

In NRPS, PKS and hybrid systems, besides the essential aforementioned catalytic domains, the presence of additional “tailoring domains”, which introduce structural modifications into the canonical building blocks and contribute to the amazing structural diversity in NRP and PK metabolites, is frequently observed. These domains can be encoded within the biosynthetic gene clusters, either fused to other catalytic domains or as self-standing domains [9]. Their activities range from hydroxylation, halogenation, methylation, racemization, heterocyclization, lipidation and glycosilation. Several PLP-dependent proteins were also identified in multimodular biosynthetic machineries, some operating as self-standing domains, others incorporated in multidomain enzymes containing at least one carrier protein. Examples of PLP-dependent enzymes postulated to be involved in the formation of building blocks are found in the biosynthesis of peptidyl nucleoside antibiotic pacidamycin: PacE and PacS belonging to the type II fold, and PacT belonging to the type I fold [11]. A PLP-dependent protein is involved in an interesting and very unusual chain releasing mechanism in the biosynthesis of the fungal polyketide mycotoxin fumonisin, namely by incorporation of two carbons and one amino group from alanine into the acyl chain [12]. A stand-alone PLP dependent enzyme, MxcL, is hypothesized to participate in the final step of the biosynthesis of myxochelin B, the catecholate siderophore produced by *Stigmatella aurantiaca* Sg a15 [13], namely in the transamination of the aldehyde group present in the late biosynthetic intermediate. The insertion of an amino group into a polyketide biosynthetic precursor by transamination of the carbonyl function is a process operating also in the biosynthesis of antimicrobial polyamino antibiotics zeamines produced by *Serratia plymuthica* RVH1 [14]. In particular, the gene *zmn12* present in the complex biosynthetic cluster of this compound, encodes a protein containing a domain with a putative aminotransferase activity, homologous to the type I PLP–dependent glutamate-1-semialdehyde aminotransferase.

The functionally characterized PLP-dependent domains belonging to fold type I which operate *in cis* within mixed NRPS/PKS multienzyme systems are those involved in the biosynthesis of the potent antifungal cyclic lipopeptide mycosubtilin [15] and of the tripyrrolic metabolite prodigiosin [16]. In the biosynthesis of mycosubilin [15], an aminotransferase domain (AMT) embedded within the PKS/NRPS hybrid enzyme MycA and located at the interface of the PKS and NRPS modules, catalyzes the incorporation of an amine group from the amine donor, Gln, into the protein-bound PLP and subsequently to the β-ketothioester tethered to the ACP domain of the polyketide moiety. A different role of a PLP domain was established in the formation of prodigiosin belonging to the family of tripyrrole red pigments prodiginines produced by *Serratia* and *Streptomyces* bacterial strains, which are attracting increasing interest because of their immunosuppressive, anticancer, antimicrobial, and antimalarial activities. The PLP-dependent domain, SerT, located on a module containing also two ACP domains, PigH, is predicted to generate a C_2_ fragment by decarboxylation of l-serine, which is then used for pyrrole B ring formation [16].

In this paper, we focus specifically on PLP-dependent domains of fold type I occurring covalently linked in multidomain frameworks related to NRPS and/or PKS-like assemblies in bacterial systems. Since the identification of these domains is relatively recent, we undertook an *in silico* analysis with the aim to contribute to the clarification of some aspects concerning their function, structural remodeling and relationship to the homologous, traditional PLP-dependent enzymes.

## Results

### Construction of the hidden Markov model representative of fold type I PLP enzymes

**Figure 1:**
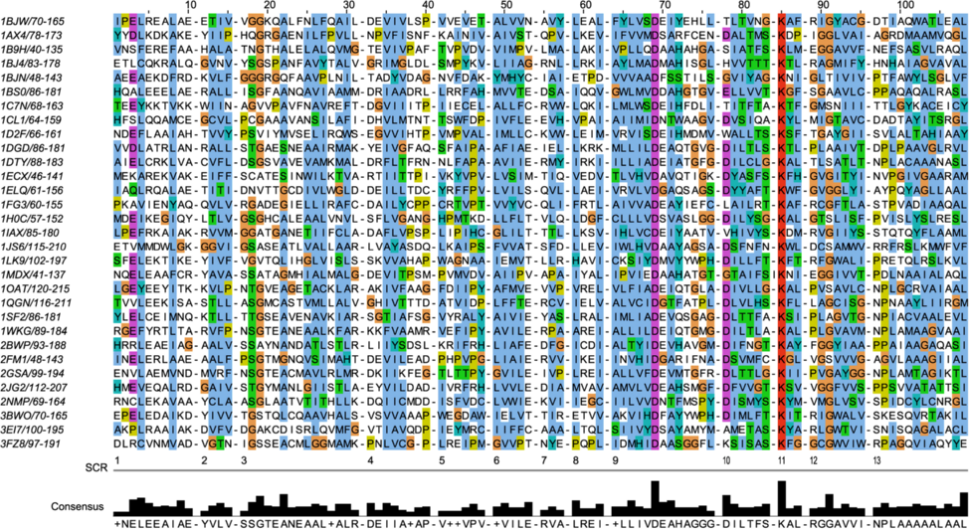
**Structurally Conserved Regions.** Alignment of the Structurally Conserved Regions (SCR) of the 31 fold type I structures considered. Colors indicate conservation of residue physico-chemical properties. Each structure is labeled by its PDB code flanked by the sequence positions encompassing the reported SCRs. “SCR line” numbers the 13 conserved regions; below is the conservation histogram and the consensus sequence. The identically conserved residues in position 69 and 85 are the Asp interacting with the cofactor pyridine nitrogen and the Lys forming the Schiff base, respectively. Indels are not shown for easing the interpretation of the figure. Indel positions are denoted by the all-gap columns separating the different SCRs.

We have expanded the non-redundant set of proteins belonging to the fold type I family (Table 1) already reported [5]. Twelve new structures were included to obtain a total of 31 fold type I proteins aligned (Figure 1). The structurally conserved regions [17] belonging to the large and small domain of the type I monomer have been identified. Since the small domain is the most variable among the fold type I proteins, the alignment portion used in the HMM profile encompasses the regions containing the major domain and the helix bridging the minor domain. This region corresponds to the first 13 SCRs. The long insertions/deletions (*indels*) have been kept in the alignment in order to confer the HMM profile ability to adequately modeling the indels expected to occur in distantly related structures. We will refer to the HMM profile calculated from this alignment as PLP_domain profile.

**Table 1:** List of structures of PLP-dependent type-I domains utilized for the calculation of the PLP_domain profile

**PDB code**^**a)**^	**Enzyme description**	**Source**	**Resolution (Å)**
1. 1AX4	Tryptophanase	*Proteus vulgaris*	2.10
2. 1B9H	3-amino-5-hydroxybenzoate synthase	*Amycolatopsis mediterranei*	2.00
3. 1BJ4	Serine hydroxymethyltransferase	*Homo sapiens*	2.65
4. 1BJN	Phosphoserine aminotransferase	*Escherichia coli*	2.29
5. 1BJW	Aspartate aminotransferase	*Thermus thermophilus*	1.80
6. 1BS0	8-amino-7-oxononanoate synthase	*Escherichia coli*	1.65
7. 1C7N	Cystalysin	*Treponema denticola*	1.90
8. 1CL1	Cystathionine beta-lyase	*Escherichia coli*	1.83
9. 1D2F	MalY protein	*Escherichia coli*	2.50
10. 1DGD	Dialkylglycine decarboxylase	*Burkholderia cepacia*	2.80
11. 1DTY	Adenosylmethionine aminotransferase	*Escherichia coli*	2.14
12. 1ECX	NifS-like protein	*Thermotoga maritima*	2.70
13. 1ELQ	L-cysteine/L-cystine C-S lyase	*Synechocystis sp.*	1.80
14. 1FG3	Histidinol-phosphate aminotransferase	*Escherichia coli*	2.20
**15. 1H0C**	Alanine-glyoxylate aminotransferase	*Homo sapiens*	2.50
**16. 1IAX**	1-aminocyclopropane-1-carboxylate synthase	*Lycopersicon esculentum*	2.80
17. 1JS6	Dopa decarboxylase	*Sus scrofa*	2.60
18. 1LK9	Alliin lyase	*Allium sativum*	1.53
19. 1MDX	ArnB aminotransferase	*Salmonella typhimurium*	1.96
**20. 1OAT**	Ornithine aminotransferase	*Homo sapiens*	2.50
21. 1QGN	Cystathionine gamma-synthase	*Nicotiana tabacum*	2.90
**22. 1SF2**	4-aminobutyrate-aminotransferase	*Escherichia coli*	2.40
**23. 1WKG**	Acetylornithine aminotransferase	*Thermus thermophilus HB8*	2.25
**24. 2BWP**	5-aminolevulinate synthase	*Rhodobacter capsulatus*	2.70
**25. 2FM1**	Threonine Aldolase	*Thermotoga maritima*	2.25
26. 2GSA	Glutamate-1-semialdehyde 2,1-aminomutase	*Synechococcus sp.*	2.40
**27. 2JG2**	Serine Palmitoyltransferase	*Pseudomonas paucimobilis*	1.30
**28. 2NMP**	Cystathionine gamma lyase	*Homo sapiens*	2.60
**29. 3BWO**	L-tryptophan aminotransferase	*Arabidopsis thaliana*	2.40
**30. 3EI7**	LL-diaminopimelate aminotransferase	*Arabidopsis thaliana*	1.99
**31. 3FZ8**	Glutamate decarboxylase beta	*Escherichia coli*	3.00

^a)^Boldfaced codes denote the new structures added to the original set.

### Detection of sequences containing the type I PLP-domains through databank searches

At the completion of the databank searches, 206 sequences were collected using the criteria and the filtering procedure reported in Methods section; a detailed list is reported in Additional file 1: Table S1.

### Phylogenic analysis of type I PLP domains embedded in the NRPS or PKS multienzyme assemblies

**Figure 2:**
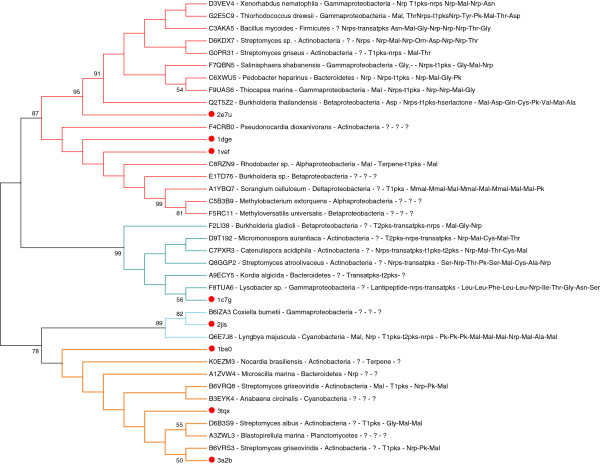
**Topology of the unrooted consensus tree calculated from the multiple alignment of the non-redundant set of type I domains.** The percentage of replicate trees in which the associated taxa clustered together in the bootstrap test (1000 replicates) is shown next to the branches whenever the value was greater than 50. Sequences are labeled by their UniProt code and the following information, in order: specie name, *phylum*, specificity, cluster type and product (definitions refers to those reported in Table 2). Question mark denotes unknown information. Red circles indicate reference structures identified by their PDB id codes: [PDB:2E7U] is glutamate-1-semialdehyde 2,1-aminomutase from *Thermus termophilus*; 1DGE, dialkylglycine decarboxylase from *Burkholderia cepacia*; [PDB:1VEF], acetylornithine aminotransferase from *Thermus termophilus*; [PDB:3A2B], serine palmitoyltransferase from *Sphyngobacterium multivorum*; [PDB:1BS0], 8-amino-7-oxononanoate synthase from *Escherichia coli*; [PDB:3TQX], 2-amino-3-ketobutyrate coenzyme A ligase from *Coxiella burnetii*; [PDB:1C7G], tyrosine phenol-lyase from *Erwinia herbicola*; [PDB:2JIS], cysteine sulfinic acid decarboxylase from *Homo sapiens.* Subtrees defining the four families are drawn with different colours. The tree is unrooted.

The sequences corresponding to the type I PLP domains were extracted from the parent sequences. A subset was selected using the routine “skipredundant” of the EMBOSS suite [18] to remove sequences sharing more than 70% identity to one of the other. Thirty sequences were retained from the initial set of 206 type I domains and were multiply aligned. Phylogenetic analyses were applied to visualize the relationships among domain families. The resulting consensus tree reported in Figure 2 suggests that the type I domains can be divided into four distinct groups.

The most numerous group corresponds to the Pfam Aminotran_3 family (code PF00202) (Table 2), related to the PLP AT-II family [19]. Members of the family are the glutamate-1-semialdehyde 2,1-aminomutase [PDB:2E7U], the acetylornithine aminotransferase [PDB:1VEF] and the 2,2-dialkylglycine decarboxylase [PDB:1DGE]. A second group matches the Pfam family Aminotran_1_2 (PF00155) structurally related to the PLP-dependent CoA family [19] to which serine palmitoyltransferase [PDB:3A2B], 2-amino-3-ketobutyrate CoA ligase [PDB:3TQX] and 8-amino-7-oxononanoate synthase [PDB:1BS0] belong. The third group coincides with the Pfam family Beta_elim_lyase (PF01212) to which tyrosine phenol-lyase [PDB:3C7G], member of the decarboxylase group I [19], belongs. The last group, the least populated, is related to human cysteine sulfinic acid decarboxylase [PDB:2JIS] and glutamic acid decarboxylase [PDB:2OKK]. The Pfam name of this group is Pyridoxal_deC (PF00282).

**Table 2:** List of non redundant NRPS/PKS proteins containing type I PLP-dependent domains of the four families

**UniProt code**	**Specie**	**B6db**^**a)**^	**Domain layout**^**b)**^	**Specificity**^**c)**^	**Cluster type**^**d)**^	**Product**^**e)**^
**Family: Aminotran_3**						
**1. A1YBQ7**	*Sorangium cellulosum*	?	CP–KS–CP–CP–ABHy6–**PLP1**	?	T1pks	Mmal-Mmal-Mal-Mmal-Mal-Mmal-Mal-Mal-Pk
**2. C3AKA5**	*Bacillus mycoides*	GSA	KS–CP–**PLP1**	?	Nrps-transatpks	Asn-Mal-Gly-Nrp-Nrp-Nrp-Thr-Gly
**3. C5B3B9**	*Methylobacterium extorquens*	TPA	CP–ABHy–**PLP1–**FMO	?	?	?
**4. C6XWU5**	*Pedobacter heparinus*	GSA	A–CP–KS–AT–CP–**PLP1**	Nrp	Nrps-t1pks	**Nrp-Mal**-Gly-Pk
**5. C8RZN9**	*Rhodobacter sp. SW2*	DAT	KS–AT–ADHN–ADHznN–KR–CP–**PLP1**	Mal	Terpene-t1pks	Mal
**6. D3VEV4**	*Xenorhabdus nematophila*	GSA	CP–**PLP1–**C–A–CP	Nrp	T1pks-nrps	Nrp-Mal-**Nrp**-Asn
**7. D6KDX7**	*Streptomyces sp. e14*	GSA	CP–**PLP1–**C	?	Nrps	Nrp-Mal-Nrp-Orn-Asp-Nrp-Nrp-Thr
**8. E1TD76**	*Burkholderia sp.*	DAT	CP–**PLP1–**SDH	?	?	?
**9. F4CRB0**	*Pseudonocardia dioxanivorans*	DOT	A–CP–**PLP1**	?	?	?
**10. F5RC11**	*Methyloversatilis universalis*	OAT	CP–ABHy6–**PLP1**–FMO	?	?	?
**11. F7QBN5**	*Salinisphaera shabanensis E1L3A*	GSA	C–A–CP–KS–AT–CP–**PLP1–**C–HxxPF–A–CP	Gly,-	Nrps-t1pks	**Gly-Mal-Nrp**
**12. F9UAS6**	*Thiocapsa marina 5811*	GSA	KS–AT–CP–**PLP1**	Mal	Nrps-t1pks	Nrp-Nrp-**Mal**-Gly
**13. G0PR31**	*Streptomyces griseus*	GSA	**PLP1**–Luc–C–HxxPF–CP–C	**?**	T1pks-nrps	Mal-Thr
**14. G2E5C9**	*Thiorhodococcus drewsii AZ1*	GSA	A–CP–KS–AT–CP–**PLP1–**C–CP–C–HxxPF–C–HxxPF–A–CP–C–HxxPF	Mal, Thr	Nrps-t1pks	Nrp-Tyr-Pk-**Mal-Thr**-Asp
**15. Q2T5Z2**	*Burkholderia thailandensis*	GSA	KS–AT–CP–**PLP1–**C–HxxPF–A–CP–C	Asp	Nrps-t1pks-hserlactone	**Mal-Asp**-Gln-Cys-Pk-Val-Mal-Ala
**Family: Aminotran_1_2**						
**1. A1ZVW4**	*Microscilla marina*	AOS	A–CP–**PLP2**	Nrp	?	?
**2. A3ZWL3**	*Blastopirellula marina*	AOS	A–CP–**PLP2**	?	?	?
**3. B3EYK4**	*Anabaena circinalis*	AOS	CP–**PLP2**	?	?	?
**4. B6VRQ8**	*Streptomyces griseoviridis*	AOS	A–CP–KS–AT–**PLP2**	Mal	T1pks	Nrp-Pk-**Mal**
**5. B6VRS3**	*Streptomyces griseoviridis*	AOS	CP–CP–**PLP2**	?	T1-pks	Nrp-Pk-Mal
**6. D6B3S9**	*Streptomyces albus J1074*	AOS	KR–CP–**PLP2**	?	T1pks	Gly-Mal-Mal
**7. K0EZM3**	*Nocardia brasiliensis*	AOS	A–CP–Luc–**PLP2**	?	Terpene	?
**Family: Beta_elim_lyase**						
**1. D9T192**	*Micromonospora aurantiaca*	TPL	CP–KR–KS–ECH–CP–KS–KR–MT–CP–KS–KR–CP–KS–CP–CP–DUF2156–**PLP3**–ABHy1	?	T2pks-nrps-transatpks	Nrp-Mal-Cys-Mal-Thr
**2. C7PXR3**	*Catenulispora acidiphila*	TPL	CP–KR–KS–ECH–CP–KS–KR–MT–CP–KS–KR–CP–KS–CP–DUF2156–**PLP3**–ABHy5	?	Nrps-transatpks-t1pks-t2pks	Nrp-Mal-Thr-Cys-Mal
**3. Q8GGP2**	*Streptomyces atroolivaceus*	TPL	CP–KR–KS–CP–KS–KR–CP–MT–CP–KS–KR–CP–KS–CP–CP–DUF2156–**PLP3**–ABHy1	?	Nrps-transatpks	Ser-Nrp-Thr-Pk-Ser-Mal-Cys-Ala-Nrp
**4. A9ECY5**	*Kordia algicida*	TPL	CP–DUF2156–**PLP3**–KS–KR–CP–KS	?	transatpks-t2pks	Pk-Mal
**5. F2LI38**	*Burkholderia gladioli*	TPL	CP–KS–KR–CP–**PLP3**–KS–CP–KS	?	T2pks-transatpks-nrps	Mal-Gly-Nrp
**6. F8TUA6**	*Lysobacter sp. ATCC 53042*	TPL	CP–DUF2156–**PLP3**–KS–KR–CP–KS–KR	?	Lantipeptide-nrps-transatpks	Leu-Leu-Phe-Leu-Leu-Nrp-Ile-Thr-Gly-Asn-Ser
**Family: Pyridoxal_deC**						
**1. Q6E7J8**	*Lyngbya majuscula*	DDC	KS–AT–ADH–KR–CP–C–HxxPF–A–**PLP4–**A–CP	Mal, Nrp	T1pks-t2pks-nrps	Pk-Pk-Pk-Mal-Mal-**Mal-Nrp**-Mal-Ala-Mal
**2. B6IZA3**	*Coxiella burnetii*	GDC	A–CP–C–**PLP4**	Gly	Nrp	Gly

^a)^Family assignment with B6db. *Abbreviations* mean: GSA = Glutamate-1-semialdehyde 2,1-aminomutase; TPA = Taurine--pyruvate aminotransferase; DAT = Diamine aminotransferase; DOT = Diaminobutyrate-2-oxoglutarate transaminase; OAT = Ornithine--oxo-acid aminotransferase; AOS = 8-amino-7-oxononanoate synthase, TPL = Tyrosine phenol-lyase; DDC = Diaminobutyrate decarboxylase; GDC = Glutamate decarboxylase. Question mark means “not assigned”.

^b)^Domain codes with Pfam id code in parentheses: A = AMP–binding enzyme (PF00501); CP = Phosphopantetheine attachment site (PF00550); KS = beta–ketoacyl synthase (PF00109); AT = Acyltransferase domain (PF00698); MT = Methyltransferase domain (PF13489); C = Condensation domain (PF00668); HxxPF = HxxPF–repeated domain (PF13745); Luc = Luciferase (PF00296); ABHyX = Alpha/beta hydrolase fold where X is the subgroup number; FMO = Flavin–containing monooxygenase (PF00743); ADHN = Alcohol dehydrogenase GroES–like domain (PF08240); ADHZnN = Zinc–binding dehydrogenase (PF00107); KR = Ketoreductase domain (PF08659); DUF4009 = Domain of unknown function (PF13193); SDH = Shikimate/quinate 5–dehydrogenase (PF01488); ECH = Enoyl–CoA hydratase/isomerase family (PF00378). Type-I domains are: PLP1 = Aminotran_3 (PF00202); PLP2 = Aminotran_1_2 (PF00155); PLP3 = Beta_elim_lyase (PF01212); PLP4 = Pyridoxal_deC (PF00282).

^c)^Predicted specificity of the AT or A domains of the chain containing the type-I domain. Nrp means generic amino acid (no consensus among the predictive methods). Question mark denotes no prediction. Mal and Pk represent malonyl and a generic polyketide, respectively.

^d)^Gene cluster type as predicted by antiSMASH. Pks and Nrps stand for Polyketide synthase and Non-ribosomal polypeptide synthetase, respectively. T1pks, T2pks, transatpks refer to Type-I, Type-II and trans-AT pks, respectively. Question mark denotes no prediction.

^e)^ Predicted cluster product. Monomers incorporated by the module containing the type-I domain are boldfaced. Question mark denotes no prediction.

The results of the annotation of the type I domain sequences through the B6 database [20] show heterogeneity with respect to the Pfam classification: the Aminotran_3 family indeed contains glutamate-1-semialdehyde 2,1-aminomutase, taurine--pyruvate aminotransferase, diamine aminotransferase, diaminobutyrate-2-oxoglutarate transaminase, ornithine--oxo-acid aminotransferase (Table 2). The Pfam families Aminotran_1_2 and Beta_elim_lyase appear homogeneous since they contain only 8-amino-7-oxononanoate synthase and tyrosine phenol lyase, respectively. The last and the less populated family Pyridoxal_deC contains, as expected, decarboxylases namely diaminobutyrate decarboxylase and glutamate decarboxylase.

A phylogenetic tree for all the collected sequences was calculated (Additional file 1: Figure S1). This tree conforms to the tree calculated for the type I sequence subset reported in Figure 2.

### Description of the domain architecture and sequence analysis of multidomain assemblies containing type I PLP enzymes

The organization and the identity of the domains contained in the parent sequences from which the type I domains were extracted, was determined through the script “Pfam_scan.pl” (see Methods). These results and those deriving from the antiSMASH [21,22] analysis are summarized in Table 2 for the non-redundant subset and in Additional file 1: Table S1 for the entire set, respectively. Apparently, Aminotran_3 family represents the vast majority of the type I domains collected. They occur almost invariantly in mixed PKS/NRPS assemblies.

Regarding the family denoted by the Pfam tag Aminotran_1_2, it can be noted that many assemblies do not contain more than three domains. However, they are very likely involved in pathways related to NRPS or PKS because the corresponding coding sequences are often adjacent to those characteristic of such biosynthetic clusters (results not shown). For example, the coding sequence [UniProt: A0SZ00] (Additional file 1: Table S2) from *Janthinobacterium lividum* [GenBank:ABK64042], is located between the sequences [GenBank:ABK64060] and [GenBank:ABK64039], corresponding to a putative peptidyl carrier protein and a putative L-prolyl-AMP-ligase, respectively.

The Beta_elim_lyase domains occur in predicted mixed PKS/NRPS transacting clusters. Interestingly, the type I domains are incorporated in modules missing any A or AT domain. This situation is reminiscent of the cluster involved in the biosynthesis of the mixed NRP/PK metabolite leinamycin from *Streptomyces atroolivaceus* S-140 [23] where the gene *lnmJ* encodes six PKS modules lacking the AT domains and a domain homologous to tyrosine phenol-lyases ([UniProt: Q8GGP2] in Table 2). It was also experimentally proved that the missing activities were provided by a discrete AT enzyme that loads the extender units *in trans*[23,24]. Moreover, we found a similar example in the mixed NRPS/PKS gene cluster 6 predicted by antiSMASH [22] analysis of the genome of the bacteria *Catenulispora acidiphila*. In this cluster, to which belongs the sequence [UniProt:C7PXR3] shown in Table 2, the occurrence of a stand-alone A domain ([UniProt:C7PXP4]) is predicted.

Pyridoxal_deC domains are the rarest since they occur only in two instances of our set, one of which could not be annotated by antiSMASH [22].

### Molecular modeling of the type I domains and docking of putative substrates

**Figure 3:**
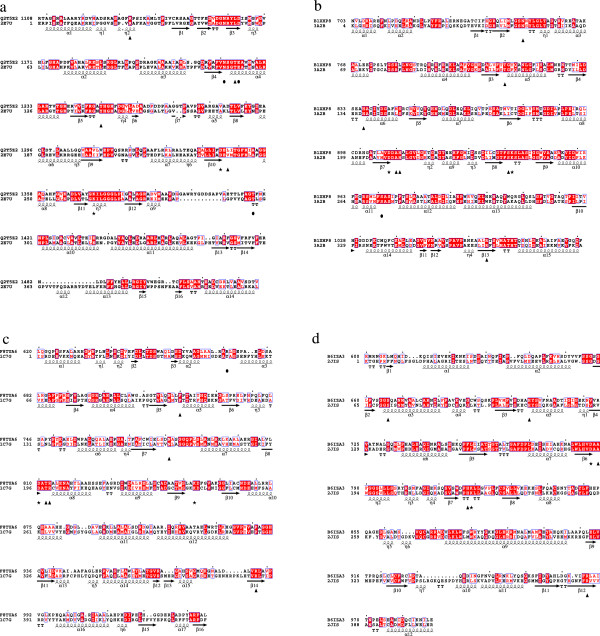
**Sequence alignment between a representative sequence of each family of type I domains and the most similar structural template.** Sequences are labeled by their databank code. Aminotran_3 **(a)**: [UniProt: Q2T5Z2] indicates polyketide synthase from *Burkholderia thailandensis*; [PDB:2E7U] is the glutamate-1-semialdehyde 2,1-aminomutase from *Thermus thermophilus HB8*. Aminotran_1_2 **(b)**: [UniProt:B1XHP8] indicates AMP-binding enzyme from *Synechococcus sp*. (strain ATCC 27264 / PCC 7002 / PR-6); [PDB:3A2B] denotes serine palmitoyltransferase from *Sphingobacterium multivorum*. Beta_elim_lyase **(c)**: [UniProt:F8TUA6] corresponds to keto-hydroxyglutarate-aldolase/polyketide synthase from *Lysobacter sp.*; [PDB:1C7G] labels the tyrosine phenol-lyase from *Erwinia herbicola*. Pyridoxal_deC **(d)**: [UniProt:B6IZA3] is the non-ribosomal peptide synthetase module from *Coxiella burnetii*; [PDB:2JIS] stands for the cysteine sulfinic acid decarboxylase from *Homo sapiens*. Secondary structures are charted below the template sequence. Helices (alpha and 3_10_ helices are designated by α or η respectively) are displayed as squiggles and beta strands (β) are rendered as arrows. Beta turns are denoted as “TT” and strict α turns as “TTT”. Dots indicate gaps. Identically conserved residues are displayed on a red background; red letters indicate conservative substitutions. Triangles mark residues known to be functionally important in the template enzyme. Black circles tag important residues from the other subunit. Stars label the Asp and the Lys residue involved in interaction with pyridine nitrogen and in Schiff-base forming, respectively. The black square in the panel **(c)** indicates the Arg381 of the template missing in the homologous PLP domain.

Homology modeling and molecular docking have been applied to map the conserved residues onto the predicted structure of a representative domain of each family and to envisage their functional role. Model-template pairs were chosen so as to maximize their percentage of sequence identity. The best Aminotran_3 pair was the PLP type I domain from polyketide synthase from *Burkholderia thailandensis* [UniProt:Q2T5Z2] and the structure of glutamate-1-semialdehyde 2,1-aminomutase from *Thermus thermophilus* (GSA) [PDB:2E7U], sharing 40% sequence identity. The Aminotran_1_2 group was represented by the type I domain from the AMP-binding enzyme from *Synechococcus* sp. [UniProt:B1XHP8] modeled onto the template serine palmitoyltransferase from *Sphingobacterium metilovorum* [PDB:3A2B]. Sequence identity shared by the two sequences was 42%. Beta_elim_lyase family was modeled using the domain from the keto-hydroxyglutarate-aldolase/polyketide synthase from *Lysobacter* sp. [UniProt:F8TUA6]. The template was the tyrosine phenol-lyase from *Erwinia herbicola* [PDB:1C7G], that shares about 24% sequence identity to the target sequence. Pyridoxal_deC family was modeled using the type I target sequence from the nonribosomal peptide synthetase module from *Coxiella burnetii* [UniProt:B6IZA3] and the template structure of human cysteine sulfinic acid decarboxylase [PDB:2JIS]. In this case, sequence identity reached 33%. Alignments used for homology modelling in each subfamily are reported in Figure 3.

**Figure 4:**
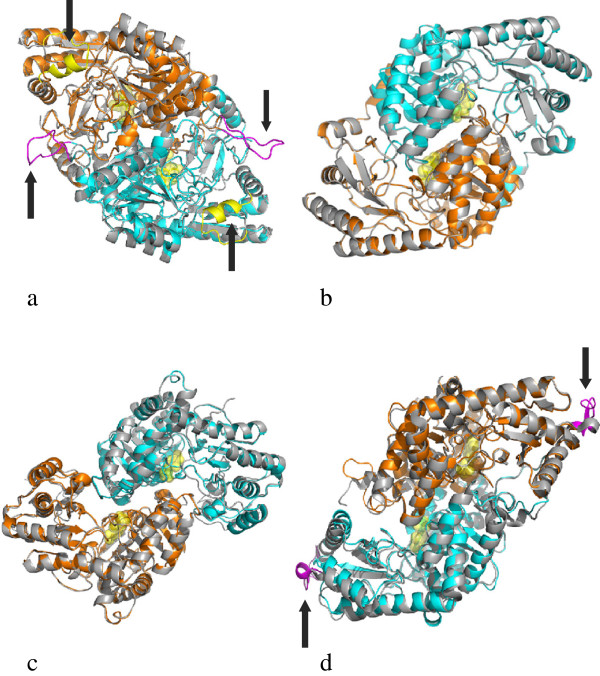
**Structural superposition of the model-template pairs.** Structural superposition of the model-template pairs for families Aminotran_3 **(a)**, Aminotran_1_2 **(b)**, Beta_elim_lyase **(c)** and Pyridoxal_deC **(d)** reported in Figure 3. Ribbon representation is used. Structural templates are colored in grey. Green and cyan indicate the model subunits. Cofactor is represented by transparent yellow spheres. Arrows point to insertion or deletion regions that are distinguished by magenta or yellow colors. The conformation of inserted magenta loop in the model of Aminotran_3 group (a) has no structural meaning: it has been modeled only with the purpose to indicate its approximate location on the protein surface.

**Figure 5:**
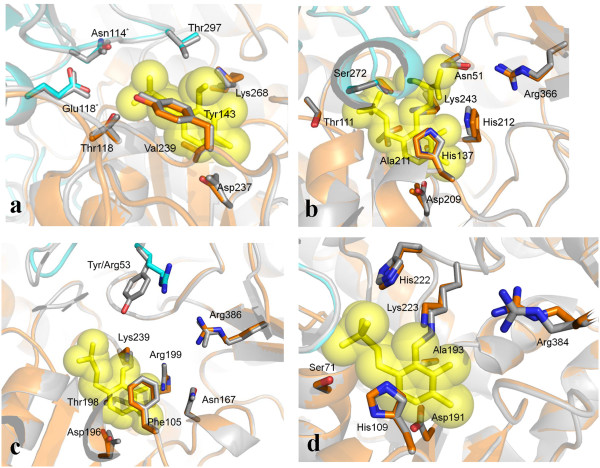
**Comparisons of the models of the active site of the domains representative of each type I subfamily.** Grey drawing indicates the reference structural template, while orange and cyan depict the two subunits of the models. Cofactor is drawn as transparent yellow spheres encapsulating stick models. Relevant side chains are rendered as sticks. Numbering refers to Figure 3. **(a)** Type I domain from polyketide synthase from *Burkholderia thailandensis* [UniProt:Q2T5Z2], and glutamate-1-semialdehyde 2,1-aminomutase from *Thermus thermophilus* HB8 (internal aldimine) [PDB:2E7U]. **(b)** AMP-binding enzyme [UniProt:B1XHP8] from *Synechococcus sp*. (strain ATCC 27264/PCC 7002/PR-6), and serine palmitoyltransferase from *Sphingobacterium multivorum* (external aldimine with serine) [PDB-3A2B]. **(c)** keto-hydroxyglutarate-aldolase/polyketide synthase from *Lysobacter sp.* [UniProt:F8TUA6] and tyrosine phenol-lyase from *Erwinia herbicola* (internal aldimine) [PDB:1C7G]. **(d)** Nonribosomal peptide synthetase module from *Coxiella burnetii* [UniProt: B6IZA3] and cysteine sulfinic acid decarboxylase from *Homo sapiens* (internal aldimine) [PDB:2JIS].

Scrutiny of the pairwise and multiple sequence alignments within the four families along with the analysis of the corresponding models, pointed out the conservation of residues functionally important in the template enzymes. In particular, the aspartate residue interacting with the pyridine nitrogen atom of pyridoxal 5′ -phosphate is identically conserved in all the four families. The lysine forming the Schiff base with the cofactor aldehydic group is also conserved, as expected, although it is replaced by the residue Thr in the domains from *Methylobacterium extorquens* [UniProt:C3B5B9] and [UniProt:H1KFY7], *Methyloversatilis universalis* [UniProt:F5RC11], and by Val in the protein from *Sorangium cellulosum* [UniProt:A1YBQ7] (Additional file 1: Figure S2). The Aminotran_3 group is characterized by a short insertion of approximately 12 residues (Figure 3a) occurring at the template positions 288–289 (the numbering system refers to the template structure). This insertion is portrayed as a loop on the surface of the model reported in Figure 4a. Likewise, the template region 364–378 corresponding to a short surface helix that contributes to the formation of the active site edge (Figure 4a), is absent in the model domain. Among the residues at the active site (Figure 5a), Tyr143 (template numbering system in Figure 3a) makes stacking interaction with the PLP ring and is conserved in many sequences of the same family. Val239, the other residue sandwiching the cofactor, is not conserved; in fact, it is replaced by the hydrophobic side chainS Ile, Leu, Ala, Met and, in three cases, by Thr (Additional file 1: Figure S2a). Several residues involved in the binding of the cofactor phosphate group are also conserved, for example Asn114, Glu118 and Thr297 from the other subunit. The functionally characterized aminotransferase domain of the enzyme MycA from *Bacillus subtilis* involved in the synthesis of the cyclic lipopeptide mycosubtilin [15], belongs to this family [UniProt :Q9R9J1]. The amine source for the MycA enzyme was proved to be the amino acid Gln. Most of the residues occurring at the active site of the model of the representative domain (Figure 5a) are identically conserved in the homologous sequences (Additional file 1: Figure S2a) suggesting that the substrate Gln may be the amine donor utilized by many of the other domains of the same Aminotran_3 family. Indeed, the docking of Gln into the active of the model (Additional file 1: Figure S3a) shows favorable interactions with the evolutionarily conserved residues observed also in the GSA template. The *α*-amino group is stabilized by an ion-pair interaction with Glu395. Thr297 of the other subunit is involved in a hydrogen bond to the carboxylate group of the substrate. Arg25 binds the carboxylic group of the substrate and is identically conserved in the polyketide synthase from *Burkholderia thailandensis* [UniProt:Q2T5Z2]. This Arg corresponds to a residue observed to be invariant in the GSA subfamily [25]; it presumably required for binding the substrate carboxylate group through a salt bridge. Finally, the *δ*-amino group of Gln forms a hydrogen bond with the conserved Ser22 residue. However, it should be noted that Arg25 and Ser22 residues occur in a non-conserved region of the type I domains where sequence alignment is intrinsically less accurate (Additional file 1: Figure S2a) and therefore the indications from the docking simulations are less reliable.

In the Aminotran_1_2 group, residues sandwiching the PLP cofactor, namely His137 and Ala211 (numbering refers to the template structure, Figure 3b), are invariant in all the homologous sequences considered (Figure 5b and Additional file 1: Figure S2b). Thr111 and Ser272 from the other subunit interact with the cofactor phosphate group and are conserved. His212 in Figure 5b is involved in the interaction with the O3 atom of PLP and is identically conserved in the homologous sequences. Regarding the binding of the substrate, it can be observed that His137 is involved in the binding of the substrate carboxylate [26]. The same role is predicted in the docked complex between substrate Ser and the homology model (Additional file 1: Figure S3b). Likewise, Arg366 should also be mentioned among the residues involved in substrate binding in the serine palmitoyltransferase template. This residue is identically conserved and it is essential for the catalysis because it forms the key PLP:l-serine quinonoid intermediate that condenses with palmitoyl-CoA [27]. Finally, according to the docked model of the PLP:l-serine quinonoid intermediate, the *β*-hydroxyl moiety of the substrate participates to a hydrogen bond with Ser242, residue conserved in all the selected members of the Aminotran_1_2 family (Additional file 1: Figure S2b).

Active site model of the type I domain representative of the Beta_elim_lyase family shows that some residues relevant for catalysis in the representative member of the group tyrosine phenol-lyase [28] are conserved. For example, Phe105 and Thr198, the residues sandwiching the PLP cofactor, are conserved (Figure 5c and Additional file 1: Figure S2c) although the latter residue is replaced by Ser in one case. The residues Arg386, Arg199 and Asn167, deemed to be involved in interaction with the carboxylic group of the reaction intermediate in tyrosine phenol-lyase, are conserved (Additional file 1: Figure S3c). On the contrary, residues that in the postulated mechanism of *β*-elimination reaction carry out the protonation of the substrate Cγ, are not present in the Beta_elim_lyase of type I embedded in multidomain context. In particular, Tyr53 of tyrosine phenol-lyase is replaced by an Arg residue in the model domain, while Arg381 that assists Tyr53 during the protonation is deleted in all the sequences reported in Additional file 1: Figure S2c, including the PLP domain involved in the biosynthesis of leinamycin [UniProt:Q8GGP2].

The Pyridoxal_deC group is the least populated; assessment of evolutionary conservation of residues possibly involved in catalysis is more difficult. His111 and Ala193 that, as in the Aminotran_1_2 family sandwich the PLP ring, are conserved. His222 and Ser71, ligands of the cofactor phosphate group, are also conserved. According to the docked model (Additional file 1: Figure S3d), the α-carboxylate group of the substrate is stabilized by an ion-pair interaction with Arg384. This residue is equivalent to Arg567 in glutamic acid decarboxylase, enzyme of the same family, that is responsible for the formation of a salt bridge to its substrate γ-aminobutyric acid [29]. Similarly Tyr253, the catalytic residue of the Group II decarboxylases that performs the protonation of the C*α* atom of the quinonoid intermediate [29], is conserved.

### Prediction of protein-protein interaction sites on the homology models of the type I domains

Prediction of the presence of potential protein-protein binding sites was carried out for the models and the relative template structures in their dimeric forms. The results suggest that some type I domains covalently incorporated in multidomain contexts possess potential protein binding sites missing in the equivalent regions of their respective structural templates. These regions are in proximity of the active sites and contain residues largely conserved in the corresponding homologs. In particular, the Aminotran_3 domain displays a small potential region encompassing the high interaction-probability residues Phe25, Ile29, Lys30, Met32, Asp380 and Gly396 (positions are relative to the template numbering system in Figure 3a). The first four residues are located in the poorly conserved N-terminal region (Additional file 1: Figure S2a). Along with the other potentially interacting sites, they form a region around the active site mouth. The Aminotran_1_2 domain shows a surface region predicted as potential protein-protein interaction site that includes Asp333 and the sequence Ala353-Lys362 (the positions refer to the template numbering system in Figure 3b). This region is located on the rim of the active site. However, the potentially interacting surface of the model is, in this case, similar to that observed in the structural template (Additional file 1: Figure S4b). The putative interaction between the CP and the type I domains of polyketide synthase from *Burkholderia thailandensis* [UniProt:Q2T5Z2] have been predicted by protein-protein docking simulation using the ClusPro server [30]. The homology model of the CP domain encompassed by the positions 932–998 of [UniProt: Q2T5Z2] has been calculated using the templates denoted by [PDB:2EHS], [PDB:1X3O], [PDB:2QNW] and [PDB:2JU2]. Although the results deriving from the docking experiments carried out with homology models should be considered with the great caution, it is interesting to note that the ten best complexes calculated by the ClusPro analysis suggest that the CP domain may interact with the surface regions of the PLP domain predicted as potential protein-protein interaction sites (Additional file 1: Figure S5). The Beta_elim_lyase model possesses a wide surface predicted as a potential interaction site, significantly larger than that predicted in the corresponding structural template (Additional file 1: Figure S4c). The interacting surface clusters into two patches located in the proximity of the active site. The first cluster is centered on the residues Arg343, His420, Gly423, Gly424, Pro431 and Tyr432. The second cluster incorporates the residues Leu104, Phe105, Pro106, Ile109 and Tyr110. The Pyridoxal_deC family, represented by the model of type I domain in the nonribosomal peptide synthetase module from *Coxiella burnetii* [UniProt:B6IZA3], displays a surface interaction propensity similar to the reference template structure of human cysteine sulfinic acid decarboxylase [PDB:2JIS] (Figure 3d). However, according to multiple sequence alignments between members of this type I domain and the other known members of the Group II decarboxylases family, the characteristic third N-terminal domain (N-domain) formed by three α-helices that fold upon dimerization [31], is lost in Pyridoxal_deC family upon incorporation in a multidomain context. It is therefore tempting to speculate that this structural deletion, which would result in an alternate entry into the active site, has evolved to accommodate the adjacent domains of the NRPS framework, *e.g.*, a phosphopantetheine binding domain, and that α-decarboxylation could occur on the amino acid substrate tethered to the phosphopantetheine arm.

## Discussion

In this work we focused specifically on the type I PLP domains involved in putative bacterial NRPS and/or PKS assemblies as tailoring domains operating in *cis*. We collected a set of predicted type I PLP sequences incorporated in multidomain environments from eubacterial sources using the HMM search using the PLP_domain profile. Only those sequences containing a phosphopantetheine binding domain (CP) were considered. In most cases, the type I domains are placed downstream from a CP domain (Additional file 1: Table S1). As a test, the selection of sequences was carried out using as a criterion the association of the type I domains with adenylation, condensation or acyltransferase domains. In all cases, only subsets of the group of sequences retrieved with the original requirement were recovered, showing the correctness of the adopted procedure. The only exception to this “rule” was the β-ketoacyl synthase from *Streptomyces violaceusniger* [UniProt:G2P368]*.* This 1136 residue long sequence is atypical since it does not possess a recognizable phosphopantetheine binding domain and displays a segment, containing about 400 residues, in the central sequence region apparently lacking any relation to known Pfam domains. In a few cases [UniProt:G8X2R2] or [UniProt:Q82RP2], Pfam annotation does not report the presence of a CP domain. However, we were able to detect those domains using the “Pfam_scan.pl” annotation script. In some cases, use of a locally installed program provides an effective way of finely tuning the parameters.

The assemblies containing the type I domains we collected, were found only in the following eubacterial *phyla*: *Acidobacteria*, *Actinobacteria, Bacteroidetes*, *Chloroflexi*, *Cyanobacteria*, *Firmicutes*, *Planctomycetes, Proteobacteria,* and *Verrucomicrobia.* However, it should be considered that the observed distribution may be significantly biased by uneven species sampling.

Sequence alignments and phylogenetic analyses indicated that four groups can be distinguished within the collected set of the type I domains. The four groups share the identical conservation (with the few exceptions reported in Results section) of the key residues Asp and Lys which are involved in interaction with the cofactor pyridine nitrogen and in the formation of a Schiff base with the aldehyde group of PLP, respectively.

We further studied the structural features characterizing these type I protein subfamilies by multiple sequence alignment, homology modeling and search of the potential binding sites possibly involved in the interaction with protein partners.

The most numerous group is the Aminotran_3 family which is structurally related to the glutamate 1-semialdehyde-2,1-aminomutase [32]. The proteins belonging to this group are predicted to occur mainly in mixed NRPS/PKS machineries. The aminotransferase domain embedded in the multidomain PKS/NRPS enzyme MycA from *Bacillus subtilis* involved in the synthesis of the cyclic lipopeptide mycosubtilin [15], is the example of the functionally characterized member of the group. The role of the aminotransferase domain is the insertion of the amino group into the polyketide biosynthetic intermediate; the amine source for the MycA enzyme was demonstrated to be glutamine [15]. A similar function was proposed also for the aminotransferase domains encoded by the genes *mxcL* and *zea12*[13,14,33] from myxochelin and zeamine biosynthetic clusters respectively, which belong to the same subfamily.

The multiple sequence alignment between a non-redundant set of sequences and the reference modeling template evidenced the structural features characteristic of these domains. As shown in Figure 3a, there is an insertion of about 12–14 residues at position 288–289 (numbering system of the structural template) that corresponds to the region 283–284 in Additional file 1: Figure S2a. The insertion is located on the surface of the domain (Figure 4a) and may be involved in modulating the interaction with the adjacent domains and/or in substrate recognition. Indeed, docking simulations suggests that a loop is involved in interaction with the CP domain (Additional file 1: Figure S5). In the surface area between the sequence positions 364–378 of the alignment in Figure 3a (corresponding to the region 364–378 in Additional file 1: Figure S2a), a deletion region is present. Further, the prediction of potential sites of protein-protein interaction assigns a significant potential to a region in the active site proximity, not visible in the equivalent position of the structural template (Additional file 1: Figure S4a). These observations, in particular the presence of a wide region of potential protein-protein interaction (Additional file 1: Figure S4b), suggest that this structural feature could be required for productive interaction with the adjacent domains in a multidomain context.

The Aminotran_1_2 group is structurally related to the Coenzyme A (CoA) family of the PLP-dependent enzymes of type I [34]. They are predicted to be involved mainly in PKS pathways. Within the CoA subfamily, serine palmitoyltransferase is the most closely related enzyme. Sequence alignments and homology modeling indicate the conservation of the residues involved in substrate-CoA interaction and the absence of long insertion/deletions with respect to the structural template (Figure 3b). Exception is the sequence of the type I domain from the *Anabaena circinalis* protein [UniProt:B3EYK4] that shows two insertions, one of which 10-residue long (Additional file 1: Figure S2b). The only experimentally characterized member of this family is the PigH protein [UniProt: Q5W247] from *Serratia marcescens*[16], involved in the biosynthesis of the three-pyrrolic red pigment prodigiosin. PigH contains two CP domains followed by a type I PLP domain, SerT (as reported in Additional file 1: Table S1), predicted to catalyze the decarboxylation of l-serine and the formation of C_2_ fragment used in the formation of the pyrrole B ring of prodigiosin. Analysis of surface of the homology model of the representative type I domain of this family suggests the presence of an increased potential for protein interaction in the proximity of the active site mouth (Additional file 1: Figure S4b). However, the increase of the interaction potential with respect to the stand-alone counterpart is less evident than in the case of the Aminotran-3 family.

Extensive remodeling of the protein surface can be conjectured also for the Beta_elim_lyase family. In fact, as in Aminotran_3 group a wide region of potential protein-protein interaction (Additional file 1: Figure S4b) is observed. The sequence divergence from the model is evident at the N-terminal part of the domain. This family is indeed characterized by domains occurring mainly at the C-terminal edge of the multidomain module. Despite the conservation of many active site key residues observed in the tyrosine phenol-lyase enzyme, some side chains involved in the catalytic mechanisms are missing. In particular, as mentioned in the Results section, Tyr53 of tyrosine phenol-lyase is replaced by an Arg residue while Arg381 is missing. Although the relevance of such variations on the functionality of these domains cannot be presently assessed, it is worth mentioning that studies on classical β-eliminating lyases showed how the mutation of the residues corresponding to Arg381 and Tyr53 affected the activity of two proteins belonging to this family. The substitution of Arg381 with Ile and Val in tyrosine phenol-lyase caused a significant impairment in the activity towards Tyr, but not in case of other substrates; moreover the same substitutions were present in the wild type and fully active tryptophan indole-lyase enzymes [35]. Similarly, the replacement of Tyr71 (corresponding to Tyr53) had different effects on the activity of the protein towards different substrates [35]. These findings indicate that Tyr53 and Arg381 are not absolutely indispensable for the catalytic activity of all the PLP enzymes of this family. Regarding the prediction of protein interaction sites on the type I domains, the results suggest the presence of a wide area possibly involved in the interaction with other protein partners as observed in other aforementioned families.

The pyridoxal_deC family, the least numerous, is related to the decarboxylase family II [19] to which the enzyme glutamate decarboxylase belongs. However, the most similar reference structure found was cysteine sulfinic acid decarboxylase, an enzyme involved in hypotaurine biosynthesis [36] which functions as an autoantigen in human endocrine autoimmune diseases [37]. Conservation of the residues essential for catalysis in glutamate decarboxylase, which is better characterized than cysteine sulfinic acid decarboxylase, suggests the possible preservation of the decarboxylase activity in this domain. The databank search showed that the pathogenic proteobacterium *Coxiella burnetii,* whose genome has been completely sequenced, possesses one of the two Pyridoxal_deC domains found during our databank searches. The other, showing high similarity with glutamate decarboxylase domains, was found in the cyanobacterium *Lyngbya majuscula,* in particular in the biosynthetic cluster of jamaicamide. Interestingly, it is embedded within the adenylation domain of the PKS/NRPS multidomain subunit JamL, but the precise function of the PLP-dependent domain in the biosynthesis of this metabolite has not been so far clarified [38].

The results of our work show that the domains belonging to the type I PLP dependent enzymes linked to a component of the multidomain frameworks related to NRPS and/or PKS-like assemblies are relatively rare but widespread among several bacterial *phyla*. These domains display conservation (except in a few cases in the Aminotran_3 family) of residues involved in cofactor binding and catalysis. However the prediction of protein-protein interaction sites suggests that the N- and C-terminal ends of the domain polypeptide chain display stronger sequence divergence with respect to the reference stand-alone structures (Additional file 1: Figure S2a). These regions are necessarily involved in connecting the linkers bridging the other domains in the multidomain subunits (Table 1). On the other hand, it must stressed that predictions of interacting sites are still very inaccurate and in this case they can be significantly biased by modeling inaccuracies especially related to the prediction of side chain solvent accessibility. Nevertheless, the differences between the prediction in the surface regions possibly involved in protein-protein interaction of the model and the templates are, at least in the case of Aminotran_3 and Aminotran_1_2 groups, very strong. These two families display the highest sequence similarity between the model and the template. Therefore, the strong differences observed can represent significant signals, while differences observed in the models of other families are less reliable. This hypothesis is supported by the results of the docking experiment carried out using the homology model of the PLP type I and CP domains of the Aminotran_3 family. Indeed, although the results of docking experiments carried out with homology models should be taken with the great caution, the ten best complexes indicate that the CP domain may interact with the surface regions of the PLP domain predicted as potential protein-protein interaction sites (Additional file 1: Figure S5).

In this context the quaternary architecture of multidomain assemblies incorporating a PLP-dependent type I domain should also be considered. Indeed, the PLP type I domains are dimers or tetramers because the proper formation of their catalytically competent active site requires the participation of residues from the adjacent subunits [39]. This structural constraint fits well with the dimeric architecture of PKS systems. On the other hand, studies with individual NRPS domains showed that they were monomers [40]; however a dimeric structure was demonstrated in the multidomain synthetase VibF and a continuum of monomeric and dimeric oligomerization states in NRPS was proposed [41]. The existence of a number of secondary metabolites of mixed PKS/NRPS origin shows that the two biosynthetic machineries are compatible and studies on multienzyme docking in hybrid megasynthetases indicated that NRPS subunits in mixed systems self-associate to interact with partner PKS homodimers [42].

## Conclusions

This work offers a systematic view of the occurrence of the type I PLP-dependent enzymes within NRPS and PKS assembly lines and predicts their structural characteristics using *in silico* methods. The results of this research contribute to a deeper understanding of the functional and structural diversity of the PLP-enzyme family of fold type I and pave the way to further studies aimed at their applications in combinatorial biosynthesis. In fact, the success in the functioning of engineered biosynthetic clusters depends, to a great extent, on efficient molecular recognition between the single components.

## Methods

### Data sources and computational tools

All sequence data processed during the work were taken from the UniProt [43] release April, 2012 or Protein Data Banks [44]. Most of the databank searches and analyses utilized the profile Hidden Markov Model (HMM) methodology as implemented in the package HMMER 3.0 [45] or relied on the BLAST suite [46]. Multiple sequence alignments were calculated either with the programs Clustal-W [47], MAFFT [48] or hmmalign [45]; sequence editing and alignment display relied on Jalview [49] or Seaview [50] editors.

### Hidden Markov model of type I PLP-enzymes

A Hidden Markov Model (HMM) of the superfamily of PLP-dependent enzymes of fold type I was calculated with the HMMER v3.0 package [45]. A profile HMM is normally calculated from a multiple alignment of a set of appropriately selected sequences belonging to the superfamily to be modeled. If the alignment is sufficiently accurate, the model should be able to recognize distantly related members of the same superfamily. Proteins belonging to the fold type I superfamily are characterized by sharing scant sequence identity, as low as 9% [5]. This characteristic provides the opportunity to design a multiple sequence alignment able to encode the structural fingerprint shared by all the members, even very distant, of the superfamily. However, accurate calculation of a multiple sequence alignment containing distant sequences is intrinsically rather difficult and strongly error-prone. This inherent difficulty has been surmounted through use of the structure-based sequence alignments [5] implemented in Combinatorial Extension [51] tool.

### Databank searches

The identification and analysis of fold type I domains within putative NRPS- or PKS-like frameworks relied on a multistep procedure:

1) The program “hmmsearch” of the HMMER v3.0 package scanned the UniProt bacterial subset (version April, 2012) using the PLP_domain query profile. The search output was filtered for the purpose of selecting multimodular sequences containing genuine PLP-domains of fold type I. As an initial and rough criterion, only hits embedded in sequences longer than 1000 residue and overlapping the query HMM profile for at least 110 residues were taken into consideration. The sequence length threshold assured that only enzymes within multidomain contexts were collected since the typical length of a stand-alone PLP enzyme is around 400 residues. The sequence coverage threshold was set taking into account that the total residues involved in the SCRs of the PLP_domain profile are 108. The limit should therefore assure presence of the SCR residues in the significant hits. Candidate sequences to be submitted to the following steps were collected;

2) The set of sequences selected at the end of step 1 was further processed by the scripts “hmmscan” in the HMMER package and “Pfam_scan.pl” available at the Pfam site [52]. The scripts compare a sequence against the Pfam database to locate known domains. Only sequences containing at least one phosphopantetheine (PP)-binding domain combined with a type I PLP-domain were retained;

3) The sequences of the type I PLP domains surviving the selection step 2, were extracted from the parent sequences according to the position boundaries assigned to them by the Pfam models. The excised sequences were multiply aligned using the program hmmalign [45] and a new HMM profile was calculated. This profile is, by definition, specific for recognizing type I domains embedded in multidomain environments containing at least one PP-binding motif. A new search has been carried with this query profile;

4) Search output was filtered with the following criteria: hits embedded in sequences longer than 500 residues and overlapping the query profile at least 110 residues, were retained; domain labeling was again carried out and only sequences characterized by the co-presence of PP-binding and type I domains were considered. The minimum length was set taking into consideration that the sum of the lengths of a typical CP domain and a typical type I enzymes is about 500 residues.

### Domain annotation and assembly characterization

The organization and the identity of the domains contained in the parent sequences from which the type I domains were extracted, were determined through annotation by means of the script Pfam_scan.pl. Gene cluster identity, substrate specificity and product prediction were carried out with the software pipeline antiSMASH v2 [22]. The type I domains isolated from the parent sequences were also compared to the families contained in the B6 database and assigned to one of them [20].

### Phylogenetic analyses

Phylogenetic analyses were carried out with the software MEGA v5.1 [53]. The Minimum Evolution method [54] implemented in the program was applied. Evolutionary distances were computed using the JTT matrix-based method [55] and all positions containing gaps and missing data were eliminated. A Bootstrap test with 1000 replicates was used to assess the predicted topology of the resulting trees. All the calculated trees were unrooted.

### Protein structure analysis, modelling and docking

Protein structure superposition and inspection utilized the Combinatorial Extension [51] and PyMOL graphics program [56], respectively. Most of the data handling was carried out with Python or Perl scripts under the Linux environment or utilized routines of the EMBOSS suite [18]. Homology modeling made use of Modeller v.9.9 [57] and PyMod [58]; models were validated with standard programs such as ProsaII [59] and Procheck [60]. Candidate templates for homology modeling were assessed through the Phyre v2.0 server for protein fold recognition [61]. Molecular docking relied on Molegro Virtual Docker [62]. Candidate molecules were prepared by adding explicit hydrogens, charges, and flexible torsions. The side-chains of the active-site residues were kept fixed during docking. A spherical energy grid with a 15 Å radius, centered on the carboxylate moiety of the aspartate residue interacting with the pyridine nitrogen atom of pyridoxal 5′-phosphate, and a grid resolution of 0.30 Å was used. Other parameters were set at their default values: scoring function, MolDock score; search algorithm: MolDock SE; number of runs: 10; maximum iterations: 1500; maximum population size, 50; maximum number of poses returned, 5; cluster similar poses with RMSD threshold: 1.00 Å. Only the highest scoring pose, according to the MolDock scheme, was kept. ESPript [63] produced figures of sequence alignments while PyMOL [56] was the program for protein structure analysis and figure design. Prediction of the potential presence of protein-protein interaction sites was carried out with the consensus method implemented in meta-PPISP at the web site http://pipe.scs.fsu.edu/meta-ppisp[64]. Protein-protein docking was carried out using the ClusPro method available at the server http://cluspro.bu.edu[30].

## Competing interests

Authors declare that there is no competing financial or non-financial interest in relation to the manuscript and its content.

## Authors’ contributions

TM carried out the computational analyses and data evaluation and participated in the study design and manuscript drafting. AP participated in data analysis, docking experiments and interpretation, and manuscript drafting. IG took part in the study design and manuscript drafting. SP conceived of the study, participated in its design, coordinated the research and drafted the manuscript. All authors read and approved the final manuscript.

## Supplementary Material

Additional file 1: Table S1 List of sequences of NRPS/PKS containing a type I domain. **Scheme 1.** Simplified scheme of typical reactions catalyzed by PLP-dependent enzymes. **Figure S1.** Topology of the unrooted consensus tree calculated from the multiple alignment of the entire set of type-I domains. **Figure S2.** Multiple alignments of the non-redundant set of sequences belonging to the three groups. **Figure S3.** Docking of putative substrates into the active site of the homology models of the type-I domains representative of each group. **Figure S4.** Prediction of the protein-protein interaction sites through the server meta-PPISP. **Figure S5.** Protein docking results obtained from the ClusPro server. Click here for file
