# Supplementary material for: Type I pyridoxal 5′-phosphate dependent enzymatic domains embedded within multimodular nonribosomal peptide synthetase and polyketide synthase assembly lines
Source: BMC Struct Biol. 2013 Oct 23;13:26. doi: 10.1186/1472-6807-13-26 (PMC3870968; doi:10.1186/1472-6807-13-26)

# Additional File 1

# Type I pyridoxal 5'-phosphate dependent enzymatic domains embedded within multimodular nonribosomal peptide synthetase and polyketide synthase assembly lines

**Teresa Milano, Alessandro Paiardini, Ingeborg Grgurina and Stefano Pascarella[§]**

Dipartimento di Scienze Biochimiche "A. Rossi Fanelli", Sapienza - Università di Roma, 00185 Roma, Italy

[§]Corresponding author

Stefano Pascarella

Dipartimento di Scienze Biochimiche

Università La Sapienza – 00185 Roma, Italy

e-mail: Stefano.Pascarella@uniroma1.it

Tel: +39 06 49917694

Fax: +39 06 49917566

Email addresses:

TM: teresa.milano@gmail.com

AP: alessandro.paiardini@uniroma1.it

IG: ingeborg.grgurina@uniroma1.it

SP: stefano.pascarella@uniroma1.it

## Table S1.

## List of sequences of NRPS/PKS containing a type I domain

| | UniProt code | Interval[a)] | Species | Phylum | Domain layout[b)] | Specificity[c)] | Cluster type[d)] | Product[e)] |
|---|---|---|---|---|---|---|---|---|
| 1. | A0YGI6 | 1777-2097 | *Marine gamma* | *Gammaproteobacteria* | A-CP-KS-AT-CP-**PLP1** | Val | Nrps-t1pks | **Val-Mal**-Gly-Nrp |
| 2. | A0ZEC0 | 1548-1882 | *Nodularia spumigena* | *Cyanobacteria* | KS-AT-CP-MT_12-**PLP1**-C-HxxPF-A-CP-C-HxxPF | Glu | Nrps-t1pks | Nrp-Mal-Pk-Mal-**Mal-Glu** |
| 3. | A1YBQ7 | 1810-2096 | *Sorangium cellulosum* | *Deltaproteobacteria* | CP-KS-CP-CP-ABHy_6-**PLP1** | ? | T1pks | Mmal-Mmal-Mal-Mmal-Mal-Mmal-Mal-Mal-Pk |
| 4. | A3NK01 | 1207-1535 | *Burkholderia pseudomallei* | *Betaproteobacteria* | KS-AT-CP-**PLP1**-C-HxxPF-A-CP-C | Asp | Nrps-t1pks-hserlactone | **Mal-Asp**-Gln-Cys-Pk-Val-Gly-Mal-Ala |
| 5. | A3P5L9 | 1198-1526 | *Burkholderia pseudomallei* | *Betaproteobacteria* | KS-AT-CP-**PLP1**-C-HxxPF-A-CP-C | Asp | Nrps-t1pks-hserlactone | **Asp**-Gln-Cys-Pk-Val-Gly-Mal-Ala |
| 6. | A4LVG4 | 1192-1520 | *Burkholderia pseudomallei* | *Betaproteobacteria* | KS-AT-CP-**PLP1**-C-HxxPF-A-CP-C | Asp | Nrps-t1pks | **Mal-Asp** |
| 7. | A6E8C2 | 1788-2123 | *Pedobacter sp.* | *Bacteroidetes* | A-CP-KS-AT-CP-**PLP1** | Nrp | Nrps-t1pks | **Nrp-Mal**-Asp |
| 8. | A6EWZ2 | 1786-2106 | *Marinobacter algicola* | *Gammaproteobacteria* | A-CP-KS-AT-CP-**PLP1** | Val | Nrps-t1pks | **Val-Mal**-Gly |
| 9. | A6P631 | 1139-1471 | *Microcystis aeruginosa* | *Cyanobacteria* | KS-AT-CP-**PLP1**-MT_12-C-HxxPF | Mal | Nrps-t1pks | Nrp-Tyr-Asp-**Mal**-Nrp |
| 10. | A7Z5A5 | 1539-1877 | *Bacillus amyloliquefaciens* | *Firmicutes* | A-CP-KS-CP-**PLP1**-C-CP-C-HxxPF-A-CP-C-HxxPF | Asn | Nrps-transatpks | Glu-Orn-Tyr-Thr-Glu-Val-Pro-Glu-Tyr-Ile-Nrp-Mal-**Pk-Asn**-Tyr-Asn-Pro-Glu-Ser-Thr |
| 11. | A8HL10 | 1212-1540 | *Burkholderia pseudomallei* | *Betaproteobacteria* | KS-AT-CP-**PLP1**-C-HxxPF-A-CP-C | Asp | Nrps-t1pks | **Mal-Asp**-Cys-Pk |
| 12. | A8TVJ3 | 1605-1923 | *Alpha proteobacterium* | *Alphaproteobacteria* | KS-AT-MT_12-CP-**PLP1**-C-CP-(C-HxxPF-A-CP)$_3$-C-HxxPF-CP-C-HxxPF-A-CP-C-HxxPF | Asn | Nrps-t1pks | Gly-Ala-Tyr-Tyr-**Mal-Asn-Val-Nrp-Gly**-Pk-Mal |
| 13. | A8UNR3 | 1706-2029 | *Flavobacteriales bacterium* | *Bacteroidetes* | A-CP-KS-AT-CP-**PLP1** | Nrp | Nrps-t1pks | **Nrp-Mal**-Gly |
| 14. | A8YJV9 | 1553-1887 | *Microcystis aeruginosa* | *Cyanobacteria* | KS-AT-CP-MT_12-**PLP1**-C-HxxPF-A-CP-C-HxxPF | Glu | Nrps-t1pks | Nrp-Mal-**Mal-Glu**-Mal-Ser-Ala-Leu-Nrp-Arg |
| 15. | A9B7X2 | 1203-1540 | *Herpetosiphon aurantiacus* | *Chloroflexi* | CP-KS-AT-CP-**PLP1**-C-HxxPF-A-MT_12-CP | Mal, Nrp | Nrps-t1pks-lantipeptide | Pk-**Mal-Nrp**-Asn-Thr-Ser-Mal-Orn-Gly-Pk-Nrp-Nrp-Asp-Asp-Ala-Val-Val-Ser-Nrp-Ser-Gly-Nrp |
| 16. | B0JPV9 | 1553-1887 | *Microcystis aeruginosa* | *Cyanobacteria* | KS-AT-CP-MT_12-**PLP1**-C-HxxPF-A-CP-C-HxxPF | Glu | Nrps-t1pks | Nrp-Mal-**Mal-Glu**-Mal-Ser-Ala-Arg-Nrp-Arg |
| 17. | B1W2F0 | 155-511 | *Streptomyces griseus* | *Actinobacteria* | **PLP1**-Luc-C-HxxPF-CP-C | **?** | T1pks-nrps | Mal-Thr |
| 18. | B2IHH5 | 1241-1569 | *Beijerinckia indica* | *Alphaproteobacteria* | KS-AT-CP-**PLP1**-C-HxxPF-A-CP | Nrp | Nrps-t1pks | Nrp-**Mal-Nrp** |
| 19. | B2IXK1 | 2321-2656 | *Nostoc punctiforme* | *Cyanobacteria* | A-Luc-CP-KS-AT-CP-**PLP1**-C-HxxPF-A-CP-C-HxxPF-A-CP-C | Orn, Phe | Bacteriocin-nrps-t1pks | Pk-**Mal-Orn-Phe**-Thr-Leu-Ala-Thr-Phe-Ile-Mal-Arg-Thr-Orn |
| 20. | B2J0F8 | 1191-1520 | *Nostoc punctiforme* | *Cyanobacteria* | KS-AT-CP-**PLP1** | Mal | Nrps-t1pks | Val-**Mal**-Val-Nrp |
| 21. | B2J0Y6 | 1075-1414 | *Nostoc punctiforme* | *Cyanobacteria* | KS-AT-**PLP1**-CP | Mal | T1pks-nrps | Pk-Asn-Mal-Asn-Asp-Mal-Thr-Mal-Nrp-**Mal**-Mal |
| 22. | B2J0Z1 | 267-601 | *Nostoc punctiforme* | *Cyanobacteria* | CP-**PLP1**-C-HxxPF-A-CP | Thr | T1pks-nrps | Pk-Asn-Mal-Asn-Asp-Mal-**Thr**-Mal-Nrp-Mal-Mal |
| 23. | B2JKM7 | 1822-2141 | *Burkholderia phymatum* | *Betaproteobacteria* | A-CP-KS-AT-CP-**PLP1**-C-HxxPF-A-CP | Val, Gly | Nrps-t1pks | **Val-Mal-Gly** |
| 24. | B2JWL0 | 112-437 | *Burkholderia phymatum* | *Betaproteobacteria* | CP-**PLP1** | ? | ? | ? |
| 25. | B3QD13 | 1200-1523 | *Rhodopseudomonas palustris* | *Alphaproteobacteria* | KS-AT-CP-**PLP1**-C-HxxPF-A-DUF4009-CP | Gly | Nrps-t1pks | Val-**Mal-Gly** |
| 26. | B3TLA1 | 1539-1877 | *Bacillus subtilis* | *Firmicutes* | A-CP-KS-CP-**PLP1**-C-CP-C-HxxPF-A-CP-C-HxxPF | Asn | Nrps-transatpks | Mal-**Pk-Asn**-Tyr-Asn-Gln-Pro-Asn-Ser |
| 27. | B4CW24 | 1-235 | *Chthoniobacter flavus* | *Verrucomicrobia* | **PLP1**-C-HxxPF-A-A-CP-TE | Val | Nrps-transatpks | Pro-**Val** |
| 28. | B6E112 | 1538-1876 | *Bacillus subtilis* | *Firmicutes* | A-CP-KS-CP-**PLP1**-C-CP-C-HxxPF-A-CP-C-HxxPF | Asn | Nrps-transatpks | Mal-**Pk-Asn**-Tyr-Asn-Ser-Glu-Ser-Thr |
| 29. | B7CEF0 | 1201-1529 | *Burkholderia pseudomallei* | *Betaproteobacteria* | KS-AT-CP-**PLP1**-C-HxxPF-A-CP-C | Asp | Nrps-t1pks | **Mal-Asp**-Gln-Cys-Val-Gly-Mal-Ala |

| | | | | | | | | |
|---|---|---|---|---|---|---|---|---|
| 30. | B8EPL1 | 1219-1556 | *Methylocella silvestris* | *Alphaproteobacteria* | KS-AT-CP-**PLP1**-C-HxxPF-A-CP | Gly | Nrps-t1pks | Val-**Mal-Gly**-Nrp |
| 31. | B8I979 | 928-1262 | *Clostridium cellulolyticum* | *Firmicutes* | KS-CP-**PLP1**-C-HxxPF-A-CP-C-A-NR-CP | Cys | Nrps-bacteriocin-transatpks-t1pks | Asn-Nrp-Mal-**Nrp-Cys**-Mal-Cys-Nrp-Nrp-Nrp |
| 32. | B9X9N2 | 1202-1537 | *Pedosphaera parvula* | *Verrucomicrobia* | KS-AT-CP-**PLP1**-C-HxxPF-A | Gly | Nrps-t1pks | Nrp-**Mal-Gly** |
| 33. | B9XPV7 | 1192-1518 | *Pedosphaera parvula* | *Verrucomicrobia* | KS-AT-CP-**PLP1**-C-HxxPF-A-CP | Ala | Nrps-t1pks | Nrp-Ala-**Mal-Ala**-Ile |
| 34. | C0J8I7 | 2575-2910 | *Burkholderia contaminans* | *Betaproteobacteria* | KS-AT-KR-CP-KS-AT-CP-**PLP1**-C-HxxPF-A-CP-C-HxxPF | Ser | Nrps-t1pks | Asn-Ser-Nrp-Nrp-Nrp-**Mal-Ser**-Pk |
| 35. | C0XWT2 | 1210-1538 | *Burkholderia pseudomallei* | *Betaproteobacteria* | KS-AT-CP-**PLP1**-C-HxxPF-A-CP-C | Asp | Nrps-t1pks | **Mal-Asp**-Gln-Cys |
| 36. | C0ZEM5 | 1581-1918 | *Brevibacillus brevis* | *Firmicutes* | A-CP-KS-CP-**PLP1**-C-CP-C-HxxPF-A-CP-C-HxxPF-A-CP-C | Orn, - | Nrps-transatpks | **Pk-Orn-Nrp**-Phe |
| 37. | C1F4N9 | 1155-1490 | *Acidobacterium capsulatum* | *Acidobacteria* | KS-AT-CP-**PLP1**-C-HxxPF-A-CP-C-HxxPF | Ala | Nrps-t1pks | Asn-Ser-Nrp-Nrp-Nrp-**Mal-Ala**-Pk |
| 38. | C3AKA5 | 881-1221 | *Bacillus mycoides* | *Firmicutes* | KS-CP-**PLP1** | ? | Nrps-transatpks | Asn-Mal-Gly-Nrp-Nrp-Nrp-Thr-Gly |
| 39. | C3U187 | 1553-1887 | *Microcystis sp.* | *Cyanobacteria* | KS-AT-CP-MT_12-**PLP1**-C-HxxPF-A-CP-C-HxxPF | Glu | Nrps-t1pks | **Mal-Glu** |
| 40. | C3U188 | 1553-1887 | *Microcystis sp.* | *Cyanobacteria* | KS-AT-CP-MT_12-**PLP1**-C-HxxPF-A-CP-C-HxxPF | Glu | Nrps-t1pks | **Mal-Glu** |
| 41. | C4I3K4 | 1198-1526 | *Burkholderia pseudomallei* | *Betaproteobacteria* | KS-AT-CP-**PLP1**-C-HxxPF-A-CP-C | ? | ? | ? |
| 42. | C5B3B9 | 815-1098 | *Methylobacterium extorquens* | *Alphaproteobacteria* | CP-ABHy_6-**PLP1**-FMO | ? | ? | ? |
| 43. | C5CQV7 | 2012-2336 | *Variovorax paradoxus* | *Betaproteobacteria* | A-CP-KS-AT-CP-**PLP1** | Val | Nrps-t1pks | **Val-Mal**-Nrp |
| 44. | C5ZLK9 | 1198-1526 | *Burkholderia pseudomallei* | *Betaproteobacteria* | KS-AT-CP-**PLP1**-C-HxxPF-A-CP-C | Asp | Nrps-t1pks-hserlactone | **Mal-Asp**-Gln-Cys-Pk-Val-Gly-Mal-Ala |
| 45. | C6U3L4 | 1201-1529 | *Burkholderia pseudomallei* | *Betaproteobacteria* | KS-AT-CP-**PLP1**-C-HxxPF-A-CP-C | Asp | Nrps-t1pk | **Mal-Asp**-Gln-Cys-Pk |
| 46. | C6XWU5 | 1730-2065 | *Pedobacter heparinus* | *Bacteroidetes* | A-CP-KS-AT-CP-**PLP1** | Nrp | Nrps-t1pks | **Nrp-Mal**-Gly-Pk |
| 47. | C7PT95 | 884-1221 | *Chitinophaga pinensis* | *Bacteroidetes* | KS-CP-**PLP1**-C-CP-C-HxxPF-A-CP-C-HxxPF-A-CP | Orn, Ala | Lantipeptide-nrps-transatpks | Pk-Nrp-Orn-Nrp-Val-Nrp-Nrp-Nrp-Asp-Orn-Asp-Asp-Mal-**Orn-Ala**-Glu-Leu-Gln |
| 48. | C8RZN9 | 2095-2437 | *Rhodobacter sp.* | *Alphaproteobacteria* | KS-AT-ADH_N-ADH_zinc_N-KR-CP-**PLP1** | Mal | Terpene-t1pks | Mal |
| 49. | C8TEL1 | 1556-1889 | *Planktothrix rubescens* | *Cyanobacteria* | KS-AT-CP-MT_12-**PLP1**-C-HxxPF-A-CP-C-HxxPF | Glu | T1pks-nrps | Nrp-Leu-Nrp-Thr-Ala-Nrp-Mal-M**al-Glu**-Pk-Mal |
| 50. | C8TEN5 | 1208-1542 | *Planktothrix rubescens* | *Cyanobacteria* | KS-AT-CP-**PLP1**-Luc-C-HxxPF-A-CP | Ser | Nrps-t1pks | **Mal-Ser**-Val-Nrp |
| 51. | D0LXU4 | 1361-1697 | *Haliangium ochraceum* | *Deltaproteobacteria* | CP-KS-AT-CP-**PLP1**-Luc-C-HxxPF-A-DUF4009-CP | Arg | Nrps-t1pks-bacteriocin | Nrp-**Mal-Arg**-Thr-Thr-Asn-Thr-Thr-Orn-Orn-Pk |
| 52. | D1RW83 | 1793-2129 | *Serratia odorifera* | *Gammaproteobacteria* | A-CP-KS-AT-CP-**PLP1**-C | Mal | Nrps-t1pks | Pk-**Mal** |
| 53. | D2QVL6 | 1831-2166 | *Spirosoma linguale* | *Bacteroidetes* | A-CP-KS-AT-CP-**PLP1** | Thr | T1pks | **Thr-Mal** |
| 54. | D3VEV4 | 324-660 | *Xenorhabdus nematophila* | *Gammaproteobacteria* | CP-**PLP1**-C-A-CP | Nrp | T1pks-nrps | Nrp-Mal-**Nrp**-Asn |
| 55. | D4HVB3 | 164-490 | *Erwinia amylovora* | *Gammaproteobacteria* | CP-**PLP1** | ? | ? | ? |
| 56. | D4IBL0 | 168-494 | *Erwinia amylovora* | *Gammaproteobacteria* | CP-**PLP1** | ? | ? | ? |
| 57. | D5MY10 | 1530-1868 | *Bacillus subtilis* | *Firmicutes* | A-CP-KS-CP-**PLP1**-C-CP-C-HxxPF-A-CP-C-HxxPF | Asn | Nrps-transatpks | Mal-Pk-Asn-Tyr-Asn-Gln-Pro-Ser-**Asn**-Nrp |
| 58. | D5X388 | 2029-2345 | *Thiomonas intermedia* | *Betaproteobacteria* | A-CP-KS-AT-CP-**PLP1** | Val | Nrps-t1pks | **Val-Mal**-Gly |
| 59. | D6AII9 | 127-454 | *Streptomyces roseosporus* | *Actinobacteria* | **PLP1**-Luc-C-HxxPF-A-CP-C-HxxPF | Nrp | T1pks-nrps | Mal-**Nrp** |
| 60. | D6CUT5 | 2042-2358 | *Thiomonas sp.* | *Betaproteobacteria* | A-CP-KS-AT-CP-**PLP1** | Val | Nrps-t1pks | **Val-Mal**-Gly |
| 61. | D6KDX7 | 252-590 | *Streptomyces sp.* | *Actinobacteria* | CP-**PLP1**-C | ? | Nrps | Nrp-Mal-Nrp-Orn-Asp-Nrp-Nrp-Thr |
| 62. | D8FTZ4 | 1215-1549 | *Oscillatoria sp.* | *Cyanobacteria* | KS-AT-CP-**PLP1** | Mal | Nrps-t1pks | Ala-Pro-**Mal** |
| 63. | D8G9C4 | 252-590 | *Oscillatoria sp.* | *Cyanobacteria* | CP-**PLP1**-C-HxxPF-A-CP | Nrp | Nrps-t1pks | Nrp-Mal-**Nrp**-Mal-Nrp-Mal-Cys-Mal-Cys-Nrp |
| 64. | D8JX17 | 1195-1528 | *Hyphomicrobium denitrificans* | *Alphaproteobacteria* | KS-AT-CP-**PLP1** | Mal | Nrps-t1pks | Thr-**Mal**-Ala-Gly |

| | | | | | | | | |
|---|---|---|---|---|---|---|---|---|
| 65. | D8NH70 | 1898-2220 | *Ralstonia solanacearum* | *Betaproteobacteria* | A-CP-KS-AT-CP-**PLP1**-Luc-(C-HxxPF-A-CP)$_4$ | Thr, Tyr, Ser,- | Nrps-t1pks | **Pk-Mal-Thr-Tyr-Ser-Nrp**-Val-Ser-Gly-Ala-Ala-Nrp |
| 66. | D9X4K3 | 115-454 | *Streptomyces viridochromogenes* | *Actinobacteria* | **PLP1**-Luc-C-HxxPF-A-CP-C-HxxPF | Nrp | Terpene-nrps-t1pks | Mal-**Nrp** |
| 67. | E0RLI8 | 1589-1928 | *Paenibacillus polymyxa* | *Firmicutes* | A-DUF4009-CP-KS-CP-**PLP1** | pK | Nrps-transatpks | Mal-**Pk**-Ser-Orn-Phe-Val-Phe-Nrp-Glu |
| 68. | E0RLI9 | 294-634 | *Paenibacillus polymyxa* | *Firmicutes* | CP-**PLP1**-C-HxxPF-CP-C-HxxPF-A-CP-C | Ser | Nrps-transatpks | Mal-**Pk-Ser**-Orn-Phe-Val-Phe-Nrp-Glu |
| 69. | E0TX30 | 1530-1868 | *Bacillus subtilis* | *Firmicutes* | A-CP-KS-CP-**PLP1**-C-CP-C-HxxPF-A-CP-C-HxxPF | Asn | Nrps-transatpk**s** | Mal-**Pk-Asn**-Tyr-Asn-Gln-Pro-Ser-Asn-Nrp |
| 70. | E1TD76 | 112-437 | *Burkholderia sp.* | *Betaproteobacteria* | CP-**PLP1** | ? | ? | ? |
| 71. | E1UV08 | 1539-1877 | *Bacillus amyloliquefaciens* | *Firmicutes* | A-CP-KS-CP-**PLP1**-C-CP-C-HxxPF-A-CP-C-HxxPF | Asn | Nrps-transatpks | Glu-Tyr-Ile-Nrp-Nrp-Mal-**Pk-Asn**-Tyr-Asn-Gln-Pro-Asn-Ser |
| 72. | E1VH58 | 334-668 | *Gamma proteobacterium* | *Gammaproteobacteria* | CP-**PLP1** | ? | ? | ? |
| 73. | E3E3Q5 | 1537-1875 | *Bacillus atrophaeus* | *Firmicutes* | A-CP-KS-CP-**PLP1**-C-CP-C-HxxPF-A-CP-C-HxxPF | Asn | Nrps-transatpks | Mal-**Pk-Asn**-Tyr-Asn-Gln-Pro-Ser-Asn |
| 74. | E3E481 | 1586-1925 | *Paenibacillus polymyxa* | *Firmicutes* | A-CP-KS-CP-**PLP1**-C-HxxPF-CP-C-HxxPF-A-CP-C | Gly | Nrps-transatpks | Gly-Orn-Gly-Ser-Phe-Ser-Orn-Orn-Ile-Nrp-Mal-**Pk-Gly**-Orn-Nrp |
| 75. | E3EE52 | 1567-1904 | *Paenibacillus polymyxa* | *Firmicutes* | A-CP-KS-CP-**PLP1**-C-CP-C-HxxPF-A-CP-C-HxxPF-A-CP-C | Tyr,Nrp | Nrps-transatpks | Mal-**Pk-Tyr-Nrp**-Ser-Ser-Ser-Ile-Ser |
| 76. | E5B1B0 | 164-490 | *Erwinia amylovora* | *Gammaproteobacteria* | CP-**PLP1** | ? | ? | ? |
| 77. | E6V5T8 | 2013-2325 | *Variovorax paradoxus* | *Betaproteobacteria* | A-CP-KS-AT-CP-**PLP1** | Val | Nrps-t1pks | **Val-Mal**-Gly |
| 78. | E6VPA4 | 1201-1521 | *Rhodopseudomonas palustris* | *Alphaproteobacteria* | KS-AT-CP-**PLP1**-C-HxxPF-A-DUF4009-CP | Gly | Nrps-t1pks | Val-**Mal-Gly** |
| 79. | E7RWH7 | 2342-2673 | *Lautropia mirabilis* | *Betaproteobacteria* | A-CP-KS-AT-CP-**PLP1** | Val | Nrps-t1pks | **Val-Mal**-Gly-Nrp |
| 80. | E8X008 | 1151-1472 | *Acidobacterium sp.* | *Acidobacteria* | KS-AT-CP-**PLP1**-C-HxxPF-A-CP | Gly | Nrps-t1pks | Thr-**Mal-Gly**-Gly |
| 81. | E8YJ66 | 112-437 | *Burkholderia sp.* | *Betaproteobacteria* | CP-**PLP1** | ? | ? | ? |
| 82. | F4CRB0 | 1648-1990 | *Pseudonocardia dioxanivorans* | *Actinobacteria* | A-CP-**PLP1** | ? | ? | ? |
| 83. | F4E4A5 | 1539-1877 | *Bacillus amyloliquefaciens* | *Firmicutes* | A-CP-KS-CP-**PLP1**-C-CP-C-HxxPF-A-CP-C-HxxPF | Asn | Nrps-transatpks | Glu-Tyr-Ile-Nrp-Mal-**Pk-Asn**-Tyr-Asn-Gln-Pro-Asn-Ser |
| 84. | F4EKV1 | 1539-1877 | *Bacillus amyloliquefaciens* | *Firmicutes* | A-CP-KS-CP-**PLP1**-C-CP-C-HxxPF-A-CP-C-HxxPF | Asn | Nrps-transatpks | Glu-Tyr-Ile-Nrp-Mal-**Pk-Asn**-Tyr-Asn-Gln-Pro-Asn-Ser |
| 85. | F4GQL5 | 1102-1429 | *Pusillimonas sp.* | *Betaproteobacteria* | KS-AT-CP-**PLP1**-C-HxxPF-A-CP-C | Nrp | Nrps-t1pks | Val-Nrp-Pk-**Mal-Nrp** |
| 86. | F5LEK1 | 1617-1956 | *Paenibacillus sp.* | *Firmicutes* | A-CP-KS-CP-**PLP1**-C-HxxPF-CP-C-HxxPF-A-CP-C | Gly | Nrps-transatpks | Mal –**Pk-Gly**-Orn-Ser-Ser-Gly-Phe-Val |
| 87. | F5RC11 | 839-1127 | *Methyloversatilis universalis* | *Betaproteobacteria* | CP-ABHy_6-**PLP1**-FMO | ? | ? | ? |
| 88. | F5SLP4 | 1570-1906 | *Desmospora sp.* | *Firmicutes* | A-CP-KS-CP-**PLP1**-C-CP-C-HxxPF | Pk | Nrps-transatpks | Pk-Pk |
| 89. | F7QBN5 | 2244-2573 | *Salinisphaera shabanensis* | *Gammaproteobacteria* | C-A-CP-KS-AT-CP-**PLP1**-C-HxxPF-A-CP | Gly,- | Nrps-t1pks | **Gly-Mal-Nrp** |
| 90. | F7TVF0 | 948-1286 | *Brevibacillus laterosporus* | *Firmicutes* | KS-CP-**PLP1**-C-A-CP-C-HxxPF-C-A-CP-C-HxxPF | -,- | Nrps-transatpks | Nrp-Mal-Gly-Nrp-Nrp-Nrp-Gly-**Nrp-Nrp**-Gly-Nrp-Nrp |
| 91. | F8CPD9 | 1252-1584 | *Myxococcus fulvus* | *Deltaproteobacteria* | CP-KS-AT-CP-**PLP1**-C-HxxPF-A-CP | Arg | Nrps-t1pks | **Mal-Arg**-Gly-Thr-Asn-Phe-Orn-Orn-Pk |
| 92. | F8TTM9 | 890-1212 | *uncultured Acidobacteria* | *Acidobacteria* | KS-CP-**PLP1**-Luc-C-HxxPF-A-CP | Asn | Nrps-t1pks | Nrp-Mal-Mal-Mal-**Asn** |
| 93. | F9UAS6 | 1160-1487 | *Thiocapsa marina* | *Gammaproteobacteria* | KS-AT-CP-**PLP1** | Mal | Nrps-t1pks | Nrp-Nrp-**Mal**-Gly |
| 94. | G0BBN8 | 1793-2129 | *Serratia sp.* | *Gammaproteobacteria* | A-CP-KS-AT-CP-**PLP1**-C | Mal | Nrps-t1pks | **Pk-Mal** |
| 95. | G0BU58 | 1793-2129 | *Serratia sp.* | *Gammaproteobacteria* | A-CP-KS-AT-CP-**PLP1**-C | Mal | Nrps-t1pks | **Pk-Mal** |
| 96. | G0C868 | 1793-2129 | *Serratia sp.* | *Gammaproteobacteria* | A-CP-KS-AT-CP-**PLP1**-C | Mal | Nrps-t1pks | **Pk-Mal** |
| 97. | G0IKW3 | 1539-1877 | *Bacillus amyloliquefaciens* | *Firmicutes* | A-CP-KS-CP-**PLP1**-C-CP-C-HxxPF-A-CP-C-HxxPF | Asn | Nrps-transatpks | Glu-Tyr-Ile-Nrp-Mal-**Pk-Asn**-Tyr-Asn-Gln-Pro-Asn-Ser |
| 98. | G0PR31 | 155-511 | *Streptomyces griseus* | *Actinobacteria* | **PLP1**-Luc-C-HxxPF-CP-C | **?** | T1pks-nrps | Mal-Thr |
| 99. | G0VUJ9 | 1567-1904 | *Paenibacillus polymyxa* | *Firmicutes* | A-CP-KS-CP-**PLP1**-C-CP-C-HxxPF-A-CP-C-HxxPF-A-CP-C | Nrp,Tyr,- | Nrps-transatpks | Mal-**Pk-Tyr-Nrp**-Ser-Ser-Ser-Ile-Ser |

| | | | | | | | | |
|---|---|---|---|---|---|---|---|---|
| 100. | G0VW55 | 1586-1925 | *Paenibacillus polymyxa* | *Firmicutes* | A-CP-KS-CP-**PLP1**-C-HxxPF-CP-C-HxxPF-A-CP-C | Gly | Nrps-transatpks | Gly-Orn-Gly-Ser-Phe-Ser-Orn-Orn-Ile-Nrp-Mal-**Pk-Gly**-Orn-Nrp |
| 101. | G2E5C9 | 1780-2117 | *Thiorhodococcus drewsii* | *Gammaproteobacteria* | A-CP-KS-AT-CP-**PLP1**-C-CP-C-HxxPF-C-HxxPF-A-CP-C-HxxPF | Mal, Thr | Nrps-t1pks | Nrp-Tyr-Pk-**Mal-Thr**-Asp |
| 102. | G3J046 | 1250-1572 | *Methylobacter tundripaludum* | *Gammaproteobacteria* | KS-AT-CP-**PLP1**-Luc-C-HxxPF-A | Mal,Asn | Nrps-t1pks | Gly-Nrp-Gly-Gly-Mal-Pk-Mal-Mal-Gln-Mal-Gly-Gln-Pro-Val-Phe-Mal-Mal-**Mal-Asn**-Mal |
| 103. | G4NWW6 | 1530-1868 | *Bacillus subtilis* | *Firmicutes* | A-CP-KS-CP-**PLP1**-C-CP-C-HxxPF-A-CP-C-HxxPF | Asn | Nrps-transatpks | Mal-Pk-Asn-Tyr-Asn-Gln-Pro-Ser-Asn-Nrp |
| 104. | G7VQJ9 | 1595-1934 | *Paenibacillus terrae* | *Firmicutes* | A-DUF4009-CP-KS-CP-**PLP1**-C-HxxPF-CP-C-HxxPF-A-CP-C | Gly | Nrps-transatpks | Mal-**Pk-Gly**-Val-Nrp |
| 105. | G9CIA2 | 1353-1687 | *Nostoc sp.* | *Cyanobacteria* | KS-AT-CP-**PLP1**-Luc-C-HxxPF-A-CP-C-C-A-CP | Gln, Gly | T1pks-nrps | Nrp-Mal-Mal-**Mal-Gln-Gly**-Pro-Phe-Ile-Pro |
| 106. | H0BIG6 | 43-399 | *Streptomyces sp.* | *Actinobacteria* | **PLP1**-Luc-C-HxxPF-CP-C | ? | Nrps | Thr |
| 107. | H0BQM0 | 1696-2015 | *Streptomyces sp.* | *Actinobacteria* | AT-CP-KS-AT-CP-**PLP1**-C-HxxPF-A-CP | Gly | Ectoine-t3pks-nrps-t1pks | **Pk-Mal-Gly**-Mal-Ile-Nrp-Mal-Nrp-Thr-Nrp |
| 108. | H0FNM7 | 1539-1877 | *Bacillus amyloliquefaciens* | *Firmicutes* | A-CP-KS-CP-**PLP1**-C-CP-C-HxxPF-A-CP-C-HxxPF | Asn | Nrps-transatpks | Glu-Orn-Tyr-Thr-Glu-Val-Pro-Glu-Tyr-Ile-Nrp-Mal-**Pk-Asn**-Tyr-Asn-Gln-Pro-Asn-Ser |
| 109. | H0TSD4 | 1204-1542 | *Bradyrhizobium sp.* | *Alphaproteobacteria* | KS-AT-CP-**PLP1**-C-HxxPF-A-CP | Gly | Nrps-t1pks | **Mal-Gly**-Thr |
| 110. | H0U6Y0 | 946-1284 | *Brevibacillus laterosporus* | *Firmicutes* | KS-CP-**PLP1**-C-A-CP-C-HxxPF-C-A-CP-C-HxxPF | Nrp | Nrps-transatpks | Nrp-Mal-Gly-Nrp-**Nrp-Nrp**-Gly-Nrp-Nrp-Gly-Nrp-Nrp |
| 111. | H1KFY7 | 815-1098 | *Methylobacterium extorquens* | *Alphaproteobacteria* | CP-ABHy_6-**PLP1**-FMO | ? | ? | ? |
| 112. | H2AJ59 | 1499-1837 | *Bacillus amyloliquefaciens* | *Firmicutes* | A-CP-KS-CP-**PLP1**-C-CP | Pk | Nrps-transatpks | Glu-Orn-Tyr-Thr-Glu-Val-Pro-Glu-Tyr-Ile-Nrp-Mal-**Pk**-Asn-Tyr-Asn-Gln-Pro-Asn-Ser |
| 113. | Q094I7 | 1220-1555 | *Stigmatella aurantiaca* | *Deltaproteobacteria* | KS-AT-CP-**PLP1**-Luc-C-HxxPF-A-CP-TE | Gly | Nrps-t1pks | Pk-**Mal-Gly** |
| 114. | Q0B1F3 | 2584-2919 | *Burkholderia ambifaria* | *Betaproteobacteria* | KS-AT-KR-CP-KS-AT-CP-**PLP1**-C-HxxPF-A-CP-C-HxxPF | Ser | Nrps-t1pks | Asn-Ser-Nr-Nrp –Nrp-**Mal-Ser**-Pk |
| 115. | Q11F61 | 1200-1526 | *Mesorhizobium sp.* | *Alphaproteobacteria* | KS-AT-CP-**PLP1**-C-HxxPF-A-CP | Gly | Nrps-t1pks | Nrp-**Mal-Gly** |
| 116. | Q12IB9 | 1804-2137 | *Shewanella denitrificans* | *Gammaproteobacteria* | A-CP-KS-AT-CP-**PLP1**-C-HxxPF-A-CP-C-HxxPF | Asp | Nrps-t1pks | **Pk-Mal-Asp**-Nrp-Phe |
| 117. | Q1D448 | 1256-1588 | *Myxococcus xanthus* | *Deltaproteobacteria* | CP-KS-AT-CP-**PLP1**-C-HxxPF-A-CP | Arg | Nrps-t1pks | **Mal-Arg**-Gly-Thr-Asn-Nrp-Orn-Orn-Pk-Pk-Ser-Val-Gly-Mal-Nrp |
| 118. | Q211N3 | 1094-1416 | *Rhodopseudomonas palustris* | *Alphaproteobacteria* | KS-AT-CP-**PLP1**-C-HxxPF-A-CP-C | Nrp | Nrps-t1pks | **Mal-Nr-P**k-Nrp-Val |
| 119. | Q2T5Z2 | 1144-1474 | *Burkholderia thailandensis* | *Betaproteobacteria* | KS-AT-CP-**PLP1**-C-HxxPF-A-CP-C | Asp | Nrps-t1pks-hserlactone | **Mal-Asp**-Gln-Cys-Pk-Val-Mal-Ala |
| 120. | Q3JM55 | 1185-1513 | *Burkholderia pseudomallei* | *Betaproteobacteria* | KS-AT-CP-**PLP1**-C-HxxPF-A-CP-C | Asp | Nrps-t1pks | **Mal-Asp**-Gln-Cys-Pk |
| 121. | Q3M3K5 | 1199-1533 | *Anabaena variabilis* | *Cyanobacteria* | KS-AT-CP-**PLP1** | Mal | Nrps-t1pks | Val-**Mal**-Val-Nrp |
| 122. | Q3M5M7 | 269-596 | *Anabaena variabilis* | *Cyanobacteria* | CP-**PLP1**-C-HxxPF-A-CP | Arg | Nrps-t1pks | Tyr-Asn-Pk-Mal-**Arg** |
| 123. | Q5NKN7 | 1539-1877 | *Bacillus subtilis* | *Firmicutes* | A-CP-KS-CP-**PLP1**-C-CP-C-HxxPF-A-CP-C-HxxPF | Asn | Nrps-transatpks | Mal-**Pk-Asn** |
| 124. | Q63L14 | 1206-1534 | *Burkholderia pseudomallei* | *Betaproteobacteria* | KS-AT-CP-**PLP1**-C-HxxPF-A-CP-C | Asp | Nrps-t1pks-hserlactone | **Mal-Asp**-Gln-Cys-Pk-Val-Gly-Mal-Ala |
| 125. | Q6N4J8 | 1200-1522 | *Rhodopseudomonas palustris* | *Alphaproteobacteria* | KS-AT-CP-**PLP1**-C-HxxPF-A-DUF4009-CP | Gly | Nrps-t1pks | Val-**Mal-Gly** |
| 126. | Q6YK41 | 1539-1877 | *Bacillus subtilis* | *Firmicutes* | A-CP-KS-CP-**PLP1**-C-CP-C-HxxPF-A-CP-C-HxxPF | Asn | Nrps-transatpks | Mal-**Pk-Asn**-Tyr-Asn-Pro-Glu-Ser-Thr |
| 127. | Q70JZ8 | 1539-1877 | *Bacillus amyloliquefaciens* | *Firmicutes* | A-CP-KS-CP-**PLP1**-C-CP-C-HxxPF-A-CP-C-HxxPF | Asn | Nrps-transatpks | Glu-Orn-Tyr-Thr-Glu-Val-Pro-Glu-Tyr-Ile-Nrp-Mal-**Pk-Asn**-Tyr-Asn-Pro-Glu-Ser-Thr |
| 128. | Q7N0G9 | 185-516 | *Photorhabdus luminescens* | *Gammaproteobacteria* | CP-**PLP1** | ? | ? | ? |
| 129. | Q7WRI6 | 1555-1890 | *Anabaena sp.* | *Cyanobacteria* | KS-AT-CP-MT_12-**PLP1**-C-HxxPF-A-CP-C-HxxPF | Glu | Nrps-t1pks | Nrp-Nrp-Nrp-Ser-Ala-Nrp-Mal-Mal-**Mal-Glu** |
| 130. | Q847C3 | 1548-1882 | *Nodularia spumigena* | *Cyanobacteria* | KS-AT-CP-MT_12-**PLP1**-C-HxxPF-A-CP-C-HxxPF | Glu | Nrps-t1pks | **Mal-Glu**-Pk-Mal-Nrp-Mal-Thr-Nrp -Nrp |
| 131. | Q8G986 | 1557-1890 | *Oscillatoria agardhii* | *Cyanobacteria* | KS-AT-CP-MT_12-**PLP1**-C-HxxPF-A-CP-C-HxxPF | Glu | T1pks-nrps | Nrp-Leu-Nrp-Ser-Ala-Nrp-Mal-**Mal-Glu**-Pk-Mal |
| 132. | Q8XS40 | 1895-2217 | *Ralstonia solanacearum* | *Betaproteobacteria* | A-CP-KS-AT-CP-**PLP1**-Luc-(C-HxxPF-A-CP)$_4$ | Thr, Tyr, Ser, - | Nrps-t1pks | Pk-**Mal-Thr-Tyr-Ser-Nrp**-Val-Ser-Gly-Ala-Ala-Nrp |
| 133. | Q93I56 | 1539-1877 | *Bacillus subtilis* | *Firmicutes* | A-CP-KS-CP-**PLP1**-C-CP-C-HxxPF-A-CP-C-HxxPF | Asn | Nrps-transatpks | Mal-**Pk-Asn**-Tyr-Asn-Gln-Pro-Asn-Ser |

| | | | | | | | | |
|---|---|---|---|---|---|---|---|---|
| 134. | Q9FDU0 | 1553-1887 | *Microcystis aeruginosa* | *Cyanobacteria* | KS-AT-CP-MT_12-**PLP1**-C-HxxPF-A-CP-C-HxxPF | Glu | T1pks-nrps | Mal-**Mal-Glu**-Nrp-Mal |
| 135. | Q9R9J1 | 1530-1868 | *Bacillus subtilis* | *Firmicutes* | A-CP-KS-CP-**PLP1**-C-CP-C-HxxPF-A-CP-C-HxxPF | Asn | Nrps-transatpks | Mal-**Pk -Asn**-Tyr-Asn-Gln-Pro-Ser-Asn-Nrp |
| 136. | Q9RNB3 | 1553-1887 | *Microcystis aeruginosa* | *Cyanobacteria* | KS-AT-CP-MT_12-**PLP1**-C-HxxPF-A-CP-C-HxxPF | Glu | Nrps-t1pks | Nrp-Mal-Mal-**Glu-Mal**-Ser-Ala-Leu-Nrp-Arg |
| 137. | A1ZVW4 | 699-1062 | *Microscilla marina* | *Bacteroidetes* | A-CP-**PLP2** | Nrp | ? | ? |
| 138. | A3ZMA9 | 2204-2548 | *Blastopirellula marina* | *Planctomycetes* | KS-AT-ADH_N-ADH_zinc_N-KR-CP-**PLP2** | Mal | T1pks | ? |
| 139. | A3ZQ92 | 755-1100 | *Blastopirellula marina* | *Planctomycetes* | A-CP-**PLP2** | ? | ? | ? |
| 140. | A3ZWL3 | 708-1050 | *Blastopirellula marina* | *Planctomycetes* | A-CP-**PLP2** | ? | ? | ? |
| 141. | A4C5W2 | 179-522 | *Pseudoalteromonas tunicata* | *Gammaproteobacteria* | CP-**PLP2** | ? | ? | ? |
| 142. | A4FHQ0 | 1085-1430 | *Saccharopolyspora erythraea* | *Actinobacteria* | KS-AT-CP-**PLP2** | Mal | Nrps-t1pks-terpene | **Mal**-Pk-Nrp-Thr-Gly-Phe-Ser-Asn-Nrp-Nrp-Nrp |
| 143. | A0SZ00 | 292-634 | *Janthinobacterium lividum* | *Betaproteobacteria* | CP-**PLP2** | ? | ? | ? |
| 144. | A9CU06 | 766-1116 | *Kordia algicida* | *Bacteroidetes* | A-CP-**PLP2** | Nrp | ? | ? |
| 145. | B1XHP8 | 743-1085 | *Synechococcus sp.* | *Cyanobacteria* | A-CP-**PLP2** | ? | ? | ? |
| 146. | B3EYF9 | 864-1221 | *Cylindrospermopsis raciborskii* | *Cyanobacteria* | CP-**PLP2** | ? | ? | ? |
| 147. | B3EYK4 | 866-1223 | *Anabaena circinalis* | *Cyanobacteria* | CP-**PLP2** | ? | ? | ? |
| 148. | B4WGT1 | 2308-2653 | *Synechococcus sp.* | *Cyanobacteria* | KS-AT-ADH_N-ADH_zinc_N-KR-CP-**PLP2** | Mal | T1pks | **Mal**-Pk-Pk-Mal |
| 149. | B6VRQ8 | 2053-2399 | *Streptomyces griseoviridis* | *Actinobacteria* | A-CP-KS-AT-CP-**PLP2** | Mal | T1pks | Nrp-Pk-**Mal** |
| 150. | B6VRS3 | 275-620 | *Streptomyces griseoviridis* | *Actinobacteria* | CP-CP-**PLP2** | ? | ? | ? |
| 151. | C1B4R7 | 1074-1419 | *Rhodococcus opacus* | *Actinobacteria* | KS-AT-CP-**PLP2** | Mal | Terpene-t1pks | Nrp-**Mal** |
| 152. | C3RVL1 | 866-1223 | *Aphanizomenon sp.* | *Cyanobacteria* | CP-**PLP2** | ? | ? | ? |
| 153. | C3RVN6 | 870-1227 | *Lyngbya wollei* | *Cyanobacteria* | CP-**PLP2** | ? | ? | ? |
| 154. | D0LTH5 | 756-1109 | *Haliangium ochraceum* | *Deltaproteobacteria* | A-CP-**PLP2** | ? | ? | ? |
| 155. | D2R0Z4 | 752-1097 | *Pirellula staleyi* | *Planctomycetes* | A-CP-**PLP2** | ? | ? | ? |
| 156. | D4TSQ4 | 864-1221 | *Raphidiopsis brookii* | *Cyanobacteria* | CP-**PLP2** | ? | ? | ? |
| 157. | D6B3S9 | 899-1240 | *Streptomyces albus* | *Actinobacteria* | KR-CP-**PLP2** | ? | T1pks | Gly-Mal-Mal |
| 158. | D6ENX9 | 1290-1635 | *Streptomyces lividans* | *Actinobacteria* | CP-KS-AT-**PLP2** | Mal | T1pks | Cys-**Mal** |
| 159. | D6ENY2 | 282-626 | *Streptomyces lividans* | *Actinobacteria* | CP-CP-**PLP2** | ? | T1pks | Cys-Mal |
| 160. | D9VFX1 | 754-1099 | *Streptomyces sp.* | *Actinobacteria* | KS-KS-CP-**PLP2** | ? | ? | ? |
| 161. | F2AMS0 | 841-1185 | *Rhodopirellula baltica* | *Planctomycetes* | A-CP-**PLP2** | ? | ? | ? |
| 162. | F3NN71 | 283-626 | *Streptomyces griseoaurantiacus* | *Actinobacteria* | CP-**PLP2** | ? | T1pks | Ala-Pk-Mal |
| 163. | F3NN73 | 1912-2257 | *Streptomyces griseoaurantiacus* | *Actinobacteria* | A-CP-KS-AT-CP-**PLP2**-EDH | Mal | T1pks | Ala-**Pk-Mal** |
| 164. | F4XTA7 | 207-552 | *Moorea producta* | *Cyanobacteria* | CP-**PLP2** | ? | ? | ? |
| 165. | F8B5B8 | 1353-1698 | *Frankia symbiont* | *Actinobacteria* | KS-AT-CP-**PLP2** | Mal | T1pks | **Mal**-Nrp |
| 166. | F8K2V9 | 795-1140 | *Streptomyces cattleya* | *Actinobacteria* | KS-CP-**PLP2** | | | |
| 167. | G0B4R5 | 288-630 | *Serratia sp.* | *Gammaproteobacteria* | CP-CP-**PLP2** | ? | ? | ? |
| 168. | G0BLL0 | 288-630 | *Serratia sp.* | *Gammaproteobacteria* | CP-CP-**PLP2** | ? | ? | ? |
| 169. | G0C0E4 | 288-630 | *Serratia sp.* | *Gammaproteobacteria* | CP-CP-**PLP2** | ? | ? | ? |

| | | | | | | | | |
|---|---|---|---|---|---|---|---|---|
| 170. | G8SFB8 | 1164-1509 | *Actinoplanes sp.* | *Actinobacteria* | KS-AT-CP-**PLP2** | Mal | T1pks | **Mal**-Nrp |
| 171. | G8X2R2 | 792-1137 | *Streptomyces cattleya* | *Actinobacteria* | KS-CP-**PLP2** | ? | Nrps-t1pks | Ser-Pro-Mal-Mal-Mmal-Mal |
| 172. | H0IQG8 | 1141-1486 | *Mycobacterium abscessus* | *Actinobacteria* | KS-AT-CP-**PLP2** | Mal | T1pks | **Mal-Pk** |
| 173. | K0EZM3 | 1079-1421 | *Nocardia brasiliensis* | *Actinobacteria* | A-CP-Luc-**PLP2** | ? | ? | ? |
| 174. | H6QZ44 | 1258-1603 | *Nocardia cyriacigeorgica* | *Actinobacteria* | KS-AT-CP-**PLP2** | Mal | T1pks | Pk-**Mal** |
| 175. | O54153 | 282-626 | *Streptomyces coelicolor* | *Actinobacteria* | CP-CP-**PLP2** | ? | ? | ? |
| 176. | O54155 | 1951-2296 | *Streptomyces coelicolor* | *Actinobacteria* | A-CP-KS-AT-CP-**PLP2** | Mal | T1pks-nrps | Cys-**Pk-Mal** |
| 177. | Q113V0 | 190-535 | *Trichodesmium erythraeum* | *Cyanobacteria* | CP-**PLP2** | ? | ? | ? |
| 178. | Q2S9J3 | 382-724 | *Hahella chejuensis* | *Gammaproteobacteria* | CP-**PLP2** | ? | ? | ? |
| 179. | Q5W247 | 280-622 | *Serratia marcescens* | *Gammaproteobacteria* | CP-CP-**PLP2** | ? | ? | ? |
| 180. | Q5W264 | 285-627 | *Serratia sp.* | *Gammaproteobacteria* | CP-CP-**PLP2** | ? | ? | ? |
| 181. | Q7UYT8 | 841-1185 | *Rhodopirellula baltica* | *Planctomycetes* | A-CP-**PLP2** | ? | ? | ? |
| 182. | Q82RP2 | 886-1231 | *Streptomyces avermitilis* | *Actinobacteria* | AT-CP-**PLP2** | Mal | ? | ? |
| 183. | A1UUY1 | 880-1180 | *Burkholderia mallei* | *Betaproteobacteria* | KR-CP-DUF2156-**PLP3**-KS-KS | ? | Transatpks-nrps-t2pks | ? |
| 184. | A3MDI3 | 941-1241 | *Burkholderia mallei* | *Betaproteobacteria* | KR-CP-DUF2156-**PLP3**-KS-KS | ? | T2pks-transatpks-nrps | ? |
| 185. | A3NJF3 | 2811-3111 | *Burkholderia pseudomallei* | *Betaproteobacteria* | CP-KS-KR-CP-**PLP3**-KS-KS | ? | T2pks-transatpks-nrps | ? |
| 186. | A3P517 | 2788-3088 | *Burkholderia pseudomallei* | *Betaproteobacteria* | CP-KS-KR-CP-**PLP3**-KS-KS | ? | T2pks-transatpks-nrps | Mal-Gly |
| 187. | A4LGP4 | 2751-3051 | *Burkholderia pseudomallei* | *Betaproteobacteria* | CP-KS-KR-CP-**PLP3**-KS-KS | ? | ? | ? |
| 188. | A5J374 | 941-1241 | *Burkholderia mallei* | *Betaproteobacteria* | KR-CP-DUF2156-**PLP3**-KS-KS | ? | ? | ? |
| 189. | A9ECY5 | 572-932 | *Kordia algicida* | *Bacteroidetes* | CP-DUF2156-**PLP3**-KS-KR-CP-KS | ? | ? | ? |
| 190. | A9JYG5 | 941-1241 | *Burkholderia mallei* | *Betaproteobacteria* | KR-CP-DUF2156-**PLP3**-KS-KS | ? | T2pks-transatpks-nrps | ? |
| 191. | B2H7N3 | 2765-3065 | *Burkholderia pseudomallei* | *Betaproteobacteria* | CP-KS-KR-CP-**PLP3**-KS-KS | ? | T2pks-transatpks-nrps | Gly |
| 192. | B7CF08 | 2767-3067 | *Burkholderia pseudomallei* | *Betaproteobacteria* | CP-KS-KR-CP-**PLP3**-KS-KS | ? | Transatpks-nrps-t2pks | Mal-Gly |
| 193. | C4AZV5 | 941-1241 | *Burkholderia mallei* | *Betaproteobacteria* | KR-CP-DUF2156-**PLP3**-KS-KS | | Transatpks | - |
| 194. | C4I303 | 2779-3079 | *Burkholderia pseudomallei* | *Betaproteobacteria* | CP-KS-KR-CP-**PLP3**-KS-KS | ? | ? | ? |
| 195. | C5ZKZ7 | 2766-3066 | *Burkholderia pseudomallei* | *Betaproteobacteria* | CP-KS-KR-CP-**PLP3**-KS-KS | ? | T2pks-transatpks-nrps | Mal-Gly |
| 196. | C6U2W3 | 2756-3056 | *Burkholderia pseudomallei* | *Betaproteobacteria* | CP-KS-KR-CP-**PLP3**-KS-KS | ? | T2pks-transatpks-nrps | Mal |
| 197. | C7PXR3 | 6491-6775 | *Catenulispora acidiphila* | *Actinobacteria* | CP-KR-KS-ECH-CP-KS-KR-MT_12-CP-KS-KR-CP-KS-CP-DUF2156-**PLP3**-ABHy5 | ? | ? | ? |
| 198. | D9T192 | 6065-6369 | *Micromonospora aurantiaca* | *Actinobacteria* | CP-KR-KS-ECH-CP-KS-KR-MT_12-CP-KS-KR-CP-KS-CP-CP-DUF2156-**PLP3**-ABHy1 | ? | T2pks-nrps-transatpks | Nrp-Mal-Cys-Mal-Thr |
| 199. | E8RXY5 | 6101-6392 | *Micromonospora sp.* | *Actinobacteria* | CP-KR-KS-ECH-CP-KS-KR-MT_12-CP-KS-KR-CP-KS-CP-CP-DUF2156-**PLP3**-ABHy1 | ? | Transatpks-nrps-t2pks | Nrp-Mal-Cys-Thr |
| 200. | F2LI38 | 2703-3028 | *Burkholderia gladioli* | *Betaproteobacteria* | CP-KS-KR-CP-**PLP3**-KS-CP-KS | ? | T2pks-transatpks-nrps | Mal-Gly-Nrp |
| 201. | F8TUA6 | 647-988 | *Lysobacter sp.* | *Gammaproteobacteria* | CP-DUF2156-**PLP3**-KS-KR-CP-KS-KR | ? | Lantipeptide-nrps-transatpks | Leu-Leu-Phe-Leu-Leu-Nrp-Ile-Thr-Gly-Asn-Ser |
| 202. | Q3JF86 | 2772-3072 | *Burkholderia pseudomallei* | *Betaproteobacteria* | CP-KS-KR-CP-**PLP3**-KS-KS | ? | ? | ? |

| | | | | | | | | |
|---|---|---|---|---|---|---|---|---|
| 203. | Q63LK9 | 2756-3056 | *Burkholderia pseudomallei* | *Betaproteobacteria* | CP-KS-KR-CP-**PLP3**-KS-KS | ? | T2pks-transatpks-nrps | Mal-Gly |
| 204. | Q8GGP2 | 6671-6966 | *Streptomyces atroolivaceus* | *Actinobacteria* | CP-KR-KS-CP-KS-KR-CP-MT_12-CP-KS-KR-CP-KS-CP-CP-DUF2156-**PLP3**-ABHy1 | ? | Nrps-transatpks | Ser-Nrp-Thr-Pk-Ser-Mal-Cys-Ala-Nrp |
| 205. | B6IZA3 | 639-928 | *Coxiella burnetii* | *Gammaproteobacteria* | A-CP-C-**PLP4** | ? | ? | ? |
| 206. | Q6E7J8 | 3094-3479 | *Lyngbya majuscula* | *Cyanobacteria* | KS-AT-ADH_N-ADHZnN-KR-CP-C-HxxPF-A-**PLP4**-A-CP | Mal, Nrp | T1pks-t2pks-nrps | Pk-Pk-Pk-Mal-Mal-**Mal-Nrp**-Mal-Ala-Mal |

[a] Sequence position of the type-I domain within the entire sequence;

[b] Domain codes with Pfam id code in parentheses: A = AMP-binding enzyme (PF00501); ABHyX = Alpha/beta hydrolase fold where X is the subgroup number; ADHN = Alcohol dehydrogenase GroES-like domain (PF08240);ADHZnN = Zinc-“binding dehydrogenase (PF00107); AT = Acyl transferase domain (PF00698); C = Condensation domain (PF00668); CP = Phosphopantetheine attachment site (PF00550); DUF2156 = Uncharacterized conserved protein (PF09924); DUF4009 = Domain of unknown function (PF13193); ECH = Enoyl-CoA hydratase/isomerase family (PF00378); EDH = NAD dependent epimerase/dehydratase family (PF01370); FMO = Flavin containing monooxygenase (PF00743);HxxPF =HxxPF repeated domain (PF13745); KR = Ketoreductase domain (PF08659); KS = beta ketoacyl synthase (PF00109); Luc = Luciferase (PF00296); MT_12 = Methyltransferase domain, class 12 (PF08242); NR = Nitroreductase family (PF00881); TE = Thioesterase domain (PF00975); SDH = Shikimate/quinate dehydrogenase (PF01488); PLP1 = Aminotran_3 (PF00202); PLP2 = Aminotran_1_2 (PF00155); PLP3 = Beta_elim_lyase (PF01212); PLP4 = Pyridoxal_deC (PF00282);;

[c] Predicted specificity of the AT or A domains of the chain containing the type-I domain. Nrp means generic aminoacid (no consensus among the predictive methods). Question mark denotes no prediction. Mal and Pk represent malonyl and a generic polyketide, respectively.

[d] Gene cluster type as predicted by antiSMASH [1]. Pks and Nrps stand for Polyketide synthase and Non-ribosomal polypeptide synthetase, respectively. T1pks, T2pks, transatpks refer to Type-I, Type-II and trans-AT pks, respectively.

[e] Predicted cluster product. Monomers incorporated by the module containing the type-I domain are boldfaced.

# Additional Figure Legends

### Scheme 1 – Simplified scheme of typical reactions catalyzed by PLP-dependet enzmes.

Structure **A** shows the internal aldimine formed by the PLP with the active site lysine of the enzyme. Reaction with an amino acid substrates leads to the formation of the external aldimine (**B**). This compound, depending on the specific enzyme, can take different pathways acting on one of the the three bonds of the $C_\alpha$ carbon. Path **C** can lead to the reaction of transamination; path **D** shows the α-decarboxylation reaction; path **E** displays the release of the whole substrate side chain and formation of glycine. Diagram **F** is an example of a reaction acting on the $C_\beta$ carbon of the side chain. The reactions catalyzed by the PLP domains found in the biosynthesis of mycosubtilin [1], zeamine [2] and myxochelin B [3], belong to path **C**, *i.e.* transamination. The reaction catalyzed by the PLP domain involved in the biosynthesis of prodigiosin [4] falls under the path **D**, *i.e.* decarboxylation.

### Figure S1 - Topology of the unrooted consensus tree calculated from the multiple alignment of the entire set of type-I domains

The percentage of replicate trees in which the associated taxa clustered together in the bootstrap test (1000 replicates) is shown next to the branches whenever the value was greater than 50. Sequences are labeled by their UniProt code and the specie name/*phylum*. Red diamonds indicate reference structures identified by their PDB id codes: 2E7U is glutamate-1-semialdehyde 2,1-aminomutase from *Thermus termophilus*; 1VEF, acetylornithine aminotransferase from *Thermus termophilus*; 1DGE, dialkylglycine decarboxylase from *Burkholderia cepacia*; 1BS0, 8-amino-7-oxononanoate synthase from Escherichia coli; 3TQX, 2-amino-3-ketobutyrate coenzyme A ligase from *Coxiella burnetii*; 1C7G, tyrosine phenol-lyase from *Erwinia herbicola;* PDB code 2JIS stands for the cysteine sulfinic acid decarboxylase from *Homo sapiens*. Subtrees defining the four families are denoted with the same colors used in main text Fig. 2. The tree is unrooted.

**Figure S2 - Multiple alignments of the non-redundant set of sequences belonging to the three groups**

Multiple alignments of the non-redundant set of sequences belonging to the groups Aminotran_3 (a), Aminotran_1_2 (b) and Beta_elim_lyase (c). Type-I domain sequences are labeled by the UniProt identification code of the parent multidomain sequence followed by the corresponding position interval. Sequences from reference structures are denoted by their PDB codes. Secondary structures are charted below the template sequence. Helices (alpha and $3_{10}$ helices are designated by $\alpha$ or $\eta$ respectively) are displayed as squiggles and beta strands ($\beta$) are rendered as arrows. Beta turns are denoted as TT letters and strict $\alpha$ turns as TTT. Dots indicate gaps. Identically conserved residues are displayed on a red background; red letters indicate conservative substitutions. Triangles mark residues known to be functionally important in the reference enzymes. Black circles tag important residues from the other subunit. Stars label the Asp and the Lys residue involved in interaction with pyridine nitrogen and Schiff-base forming respectively. The black square in the panel (c) indicates the Arg381 of the template deleted in the homologous PLP domains.

**Figure S3 - Docking of putative substrates into the active site of the homology models of the type-I domains representative of each group**

Relevant residues and interactions are rendered as cyan sticks and yellow dashes (with distances indicated), respectively. Numbering refers to Fig. 3. (a) Type-I domain from the polyketide synthase from *Burkholderia thailandensis* (UniProt code Q2T5Z2), in complex with Gln (internal aldimine); (b) AMP-binding enzyme from *Synechococcus sp.* (strain ATCC 27264 / PCC 7002/PR-6, UniProt code B1XHP8) in complex with serine (external aldimine); (c) keto-hydroxyglutarate-aldolase/polyketide synthase from *Lysobacter sp.* (UniProt code F8TUA6) in complex with tyrosine (external aldimine); (d) non-ribosomal peptide synthetase module from *Coxiella burnetii* (UniProt code B6IZA3) in complex with cysteine sulfinic acid (external aldimine).

**Figure S4 - Prediction of the protein-protein interaction sites through the server meta-PPISP**

Surface of the proteins are colored according to the local probability of being an interaction site. Color scale ranges from blue (lowest probability) to red (highest probability) color. (a) Left side: model of the type-I domain of the polyketide synthase from *Burkholderia thailandensis* (UniProt code Q2T5Z2); right side: structure of 2E7U is the glutamate-1-semialdehyde 2,1-aminomutase from *Thermus thermophilus* HB8 (PDB code 2E7U). (b) Left side: model of the type-I domain of

AMP-binding enzyme from *Synechococcus sp.* (UniProt code B1XHP8); right side: structure of serine palmitoyltransferase from *Sphingobacterium multivorum* (PDB code 3A2B). (c) Left side: model of the type-I domain from keto-hydroxyglutarate-aldolase/polyketide synthase from *Lysobacter sp.*; right panel: 1C7G, tyrosine phenol-lyase from *Erwinia herbicola*. (d) left side: model of the type-I domain of non-ribosomal peptide synthetase module from *Coxiella burnetii*; right side: 2JIS, cysteine sulfinic acid decarboxylase from *Homo sapiens*. White arrows mark the approximate position of the active sites in the reference structures.

### Figure S5 - Protein docking results obtained from the ClusPro server

Protein docking results obtained from the ClusPro server. The model of the dimeric type-I domain of the polyketide synthase from *Burkholderia thailandensis* (UniProt code Q2T5Z2) is represented as a molecular surface depicted according to the color code utilized in Fig. S4a. The homology model of the carrier protein domain from the same synthase is represented as a ribbon model. The ten best different solutions (poses) found by ClusPro describing the interaction between the carrier protein and the type-I domain are shown as superposed ribbon structures.


1. Aron ZD, Dorrestein PC, Blackhall JR, Kelleher NL, Walsh CT: **Characterization of a new tailoring domain in polyketide biogenesis: the amine transferase domain of MycA in the mycosubtilin gene cluster**. *Journal of the American Chemical Society* 2005, **127**(43):14986-14987.
2. Masschelein J, Mattheus W, Gao LJ, Moons P, Van Houdt R, Uytterhoeven B, Lamberigts C, Lescrinier E, Rozenski J, Herdewijn P *et al*: **A PKS/NRPS/FAS hybrid gene cluster from Serratia plymuthica RVH1 encoding the biosynthesis of three broad spectrum, zeamine-related antibiotics**. *PloS one* 2013, **8**(1):e54143.
3. Silakowski B, Kunze B, Nordsiek G, Blocker H, Hofle G, Muller R: **The myxochelin iron transport regulon of the myxobacterium Stigmatella aurantiaca Sg a15**. *European journal of biochemistry / FEBS* 2000, **267**(21):6476-6485.
4. Garneau-Tsodikova S, Dorrestein PC, Kelleher NL, Walsh CT: **Protein assembly line components in prodigiosin biosynthesis: characterization of PigA,G,H,I,J**. *Journal of the American Chemical Society* 2006, **128**(39):12600-12601.

# Scheme 1

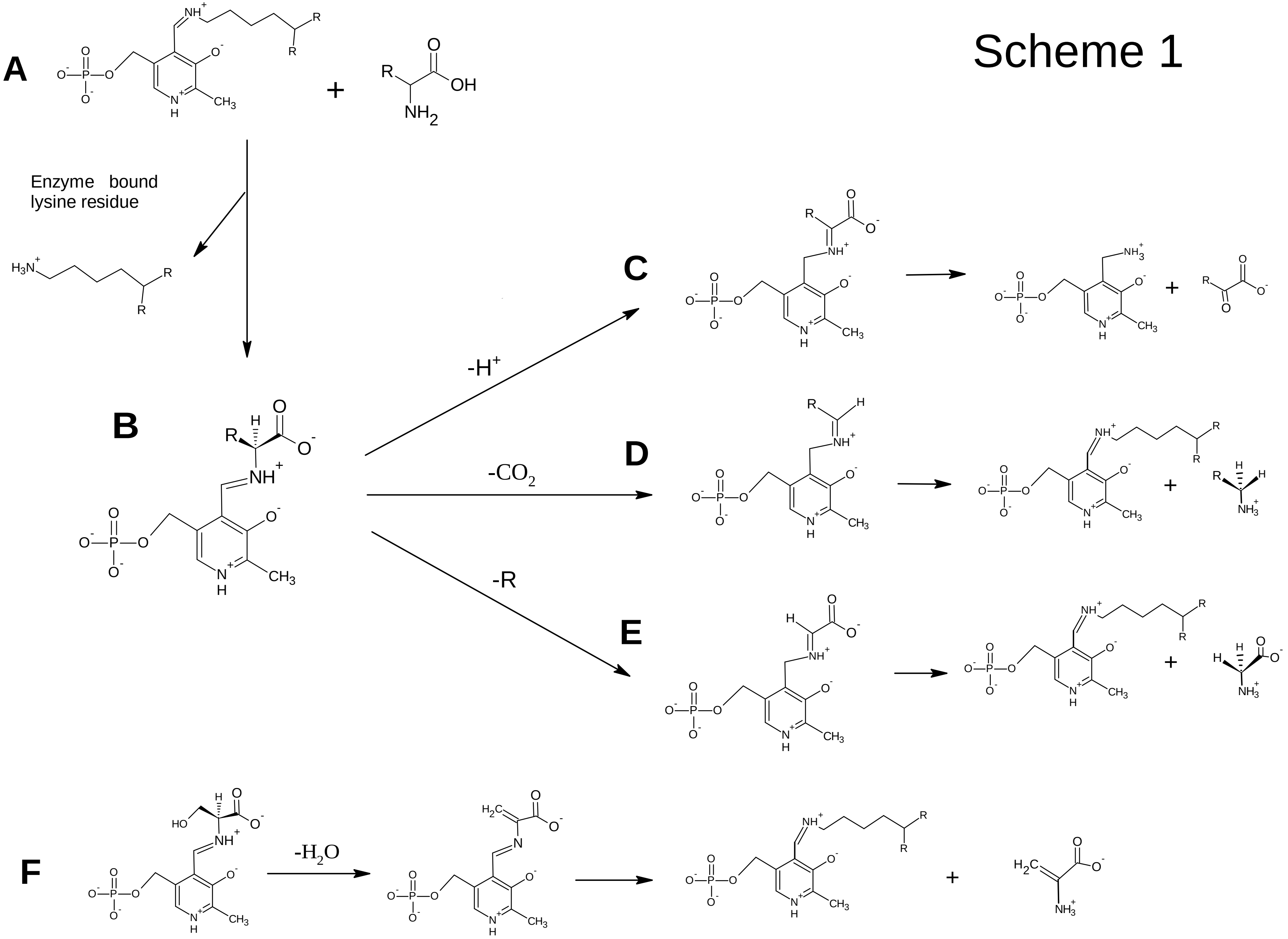

Figure S1

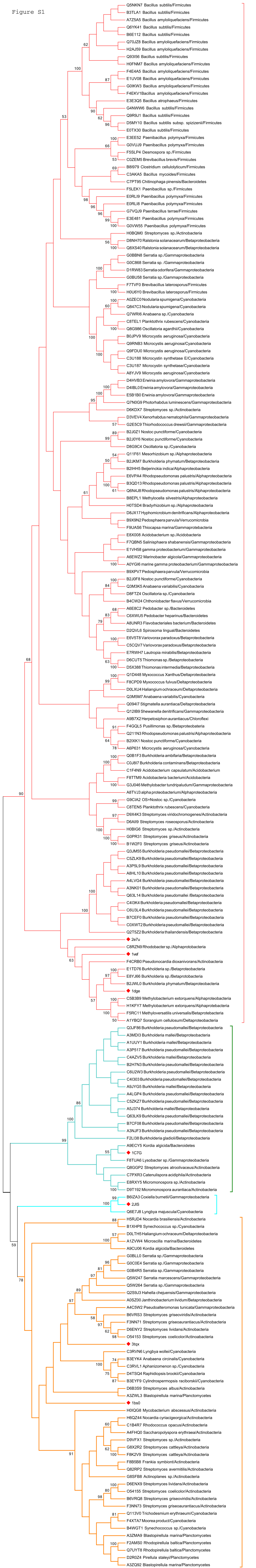

Q5NKN7 Bacillus subtilis/Firmicutes
B3TLA1 Bacillus subtilis/Firmicutes
A7Z5A5 Bacillus amyloliquefaciens/Firmicutes
Q6YK41 Bacillus subtilis/Firmicutes
B6E112 Bacillus subtilis/Firmicutes
Q70JZ8 Bacillus amyloliquefaciens/Firmicutes
H2AJ59 Bacillus amyloliquefaciens/Firmicutes
Q93I56 Bacillus subtilis/Firmicutes
H0FNM7 Bacillus amyloliquefaciens/Firmicutes
F4E4A5 Bacillus amyloliquefaciens/Firmicutes
E1UV08 Bacillus amyloliquefaciens/Firmicutes
G0IKW3 Bacillus amyloliquefaciens/Firmicutes
F4EKV1 Bacillus amyloliquefaciens/Firmicutes
E3E3Q5 Bacillus atrophaeus/Firmicutes
G4NWW6 Bacillus subtilis/Firmicutes
Q9R9J1 Bacillus subtilis/Firmicutes
D5MY10 Bacillus subtilis subsp. spizizenii/Firmicutes
E0TX30 Bacillus subtilis/Firmicutes
E3EE52 Paenibacillus polymyxa/Firmicutes
G0VUJ9 Paenibacillus polymyxa/Firmicutes
F5SLP4 Desmospora sp./Firmicutes
C0ZEM5 Brevibacillus brevis/Firmicutes
B8I979 Clostridium cellulolyticum/Firmicutes
C3AKA5 Bacillus mycoides/Firmicutes
C7PT95 Chitinophaga pinensis/Bacteroidetes
F5LEK1 Paenibacillus sp./Firmicutes
E0RLI9 Paenibacillus polymyxa/Firmicutes
E0RLI8 Paenibacillus polymyxa/Firmicutes
G7GVJ9 Paenibacillus terrae/Firmicutes
E3E481 Paenibacillus polymyxa/Firmicutes
G0VW55 Paenibacillus polymyxa/Firmicutes
H0BQM0 Streptomyces sp./Actinobacteria
D8NH70 Ralstonia solanacearum/Betaproteobacteria
Q8XS40 Ralstonia solanacearum/Betaproteobacteria
G0BBN8 Serratia sp./Gammaproteobacteria
G0C868 Serratia sp./Gammaproteobacteria
D1RW83 Serratia odorifera/Gammaproteobacteria
G0BU58 Serratia sp./Gammaproteobacteria
F7TVF0 Brevibacillus laterosporus/Firmicutes
H0U6Y0 Brevibacillus laterosporus/Firmicutes
A0ZEC0 Nodularia spumigena/Cyanobacteria
Q847C3 Nodularia spumigena/Cyanobacteria
Q7WRI6 Anabaena sp./Cyanobacteria
C8TEL1 Planktothrix rubescens/Cyanobacteria
Q8G986 Oscillatoria agardhii/Cyanobacteria
B0JPV9 Microcystis aeruginosa/Cyanobacteria
Q9RNB3 Microcystis aeruginosa/Cyanobacteria
Q9FDU0 Microcystis aeruginosa/Cyanobacteria
C3U188 Microcystin synthetase E/Cyanobacteria
C3U187 Microcystin synthetase/Cyanobacteria
A8YJV9 Microcystis aeruginosa/Cyanobacteria
D4HVB3 Erwinia amylovora/Gammaproteobacteria
D4IBL0 Erwinia amylovora/Gammaproteobacteria
E5B1B0 Erwinia amylovora/Gammaproteobacteria
Q7N0G9 Photorhabdus luminescens/Gammaproteobacteria
D6KDX7 Streptomyces sp./Actinobacteria
D3VEV4 Xenorhabdus nematophila/Gammaproteobacteria
G2E5C9 Thiorhodococcus drewsii/Gammaproteobacteria
B2J0Z1 Nostoc punctiforme/Cyanobacteria
B2J0Y6 Nostoc punctiforme/Cyanobacteria
D8G9C4 Oscillatoria sp./Cyanobacteria
Q11F61 Mesorhizobium sp./Alphaproteobacteria
B2JKM7 Burkholderia phymatum/Betaproteobacteria
B2IHH5 Beijerinckia indica/Alphaproteobacteria
E6VPA4 Rhodopseudomonas palustris/Alphaproteobacteria
B3QD13 Rhodopseudomonas palustris/Alphaproteobacteria
Q6N4J8 Rhodopseudomonas palustris/Alphaproteobacteria
B8EPL1 Methylocella silvestris/Alphaproteobacteria
H0TSD4 Bradyrhizobium sp./Alphaproteobacteria
D8JX17 Hyphomicrobium denitrificans/Alphaproteobacteria
B9X9N2 Pedosphaera parvula/Verrucomicrobia
F9UAS6 Thiocapsa marina/Gammaproteobacteria
E8X008 Acidobacterium sp./Acidobacteria
F7QBN5 Salinisphaera shabanensis/Gammaproteobacteria
E1VH58 gamma proteobacterium/Gammaproteobacteria
A6EWZ2 Marinobacter algicola/Gammaproteobacteria
A0YGI6 marine gamma proteobacterium/Gammaproteobacteria
B9XPV7 Pedosphaera parvula/Verrucomicrobia
B2JF8 Nostoc punctiforme/Cyanobacteria
Q3M3K5 Anabaena variabilis/Cyanobacteria
D8FTZ4 Oscillatoria sp./Cyanobacteria
B4CW24 Chthoniobacter flavus/Verrucomicrobia
A6E8C2 Pedobacter sp./Bacteroidetes
C6XWU5 Pedobacter heparinus/Bacteroidetes
A8UNR3 Flavobacteriales bacterium/Bacteroidetes
D2QVL6 Spirosoma linguale/Bacteroidetes
E6V5T8 Variovorax paradoxus/Betaproteobacteria
C5CQV7 Variovorax paradoxus/Betaproteobacteria
E7RWH7 Lautropia mirabilis/Betaproteobacteria
D6CUT5 Thiomonas sp./Betaproteobacteria
D5X388 Thiomonas intermedia/Betaproteobacteria
Q1D448 Myxococcus Xanthus/Deltaproteobacteria
F8CPD9 Myxococcus fulvus/Deltaproteobacteria
D0LXU4 Haliangium ochraceum/Deltaproteobacteria
Q3M5M7 Anabaena variabilis/Cyanobacteria
Q09AI7 Stigmatella aurantiaca/Deltaproteobacteria
Q12IB9 Shewanella denitrificans/Gammaproteobacteria
A9B7X2 Herpetosiphon aurantiacus/Chloroflexi
F4GQL5 Pusillimonas sp./Betaproteobacteria
Q211N3 Rhodopseudomonas palustris/Alphaproteobacteria
B2IXK1 Nostoc punctiforme/Cyanobacteria
A6P631 Microcystis aeruginosa/Cyanobacteria
Q0B1F3 Burkholderia ambifaria/Betaproteobacteria
C0J8I7 Burkholderia contaminans/Betaproteobacteria
C1F4N9 Acidobacterium capsulatum/Acidobacterium
F8TTM9 Acidobacteria bacterium/Acidobacteria
G3J046 Methylobacter tundripaludum/Gammaproteobacteria
A8TVJ3 alpha proteobacterium/Alphaproteobacteria
G9CIA2 OS=Nostoc sp./Cyanobacteria
C8TEN5 Planktothrix rubescens/Cyanobacteria
D9X4K3 Streptomyces viridochromogenes/Actinobacteria
D6AII9 Streptomyces roseosporus/Actinobacteria
H0BIG6 Streptomyces sp./Actinobacteria
G0PR31 Streptomyces griseus/Actinobacteria
B1W2F0 Streptomyces griseus/Actinobacteria
Q3JM55 Burkholderia pseudomallei/Betaproteobacteria
C5ZLK9 Burkholderia pseudomallei/Betaproteobacteria
A3P5L9 Burkholderia pseudomallei/Betaproteobacteria
A8HL10 Burkholderia pseudomallei/Betaproteobacteria
A4LVG4 Burkholderia pseudomallei/Betaproteobacteria
A3NK01 Burkholderia pseudomallei/Betaproteobacteria
Q63L14 Burkholderia pseudomallei /Betaproteobacteria
C4I3K4 Burkholderia pseudomallei/Betaproteobacteria
C6U3L4 Burkholderia pseudomallei/Betaproteobacteria
B7CEF0 Burkholderia pseudomallei/Betaproteobacteria
C0XWT2 Burkholderia pseudomallei/Betaproteobacteria
Q2T5Z2 Burkholderia thailandensis/Betaproteobacteria
2e7u
C8RZN9 Rhodobacter sp./Alphaproteobacteria
1wef
F4CRB0 Pseudonocardia dioxanivorans/Actinobacteria
E1TD76 Burkholderia sp./Betaproteobacteria
E8YJ66 Burkholderia sp./Betaproteobacteria
B2JWL0 Burkholderia phymatum/Betaproteobacteria
1dge
C5B3B9 Methylobacterium extorquens/Alphaproteobacteria
H1KFY7 Methylobacterium extorquens/Alphaproteobacteria
F5RC11 Methyloversatilis universalis/Betaproteobacteria
A1YBQ7 Sorangium cellulosum/Deltaproteobacteria
Q3JF86 Burkholderia pseudomallei/Betaproteobacteria
A3MDI3 Burkholderia mallei/Betaproteobacteria
A1UUY1 Burkholderia mallei/Betaproteobacteria
A3P517 Burkholderia pseudomallei/Betaproteobacteria
C4AZV5 Burkholderia mallei/Betaproteobacteria
B2H7N3 Burkholderia pseudomallei/Betaproteobacteria
C6U2W3 Burkholderia pseudomallei/Betaproteobacteria
C4I303 Burkholderia pseudomallei/Betaproteobacteria
A9JYG5 Burkholderia mallei/Betaproteobacteria
A4LGP4 Burkholderia pseudomallei/Betaproteobacteria
C5ZKZ7 Burkholderia pseudomallei/Betaproteobacteria
A5J374 Burkholderia mallei/Betaproteobacteria
Q63LK9 Burkholderia pseudomallei/Betaproteobacteria
B7CF08 Burkholderia pseudomallei/Betaproteobacteria
A3NJF3 Burkholderia pseudomallei/Betaproteobacteria
F2LI38 Burkholderia gladioli/Betaproteobacteria
A9ECY5 Kordia algicida/Bacteroidetes
1C7G
F8TUA6 Lysobacter sp./Gammaproteobacteria
Q8GGP2 Streptomyces atroolivaceus/Actinobacteria
C7PXR3 Catenulispora acidiphila/Actinobacteria
E8RXY5 Micromonospora sp./Actinobacteria
D9T192 Micromonospora aurantiaca/Actinobacteria
B6IZA3 Coxiella burnetii/Gammaproteobacteria
2JIS
Q6E7J8 Lyngbya majuscula/Cyanobacteria
H5RJD4 Nocardia brasiliensis/Actinobacteria
B1XHP8 Synechococcus sp./Cyanobacteria
D0LTH5 Haliangium ochraceum/Deltaproteobacteria
A1ZVW4 Microscilla marina/Bacteroidetes
A9CU06 Kordia algicida/Bacteroidetes
G0BLL0 Serratia sp./Gammaproteobacteria
G0C0E4 Serratia sp./Gammaproteobacteria
G0B4R5 Serratia sp./Gammaproteobacteria
Q5W247 Serratia marcescens/Gammaproteobacteria
Q5W264 Serratia sp./Gammaproteobacteria
Q2S9J3 Hahella chejuensis/Gammaproteobacteria
A0SZ00 Janthinobacterium lividum/Betaproteobacteria
A4C5W2 Pseudoalteromonas tunicata/Gammaproteobacteria
B6VRS3 Streptomyces griseoviridis/Actinobacteria
F3NN71 Streptomyces griseoaurantiacus/Actinobacteria
D6ENY2 Streptomyces lividans/Actinobacteria
O54153 Streptomyces coelicolor/Actinobacteria
3tqx
C3RVN6 Lyngbya wollei/Cyanobacteria
B3EYK4 Anabaena circinalis/Cyanobacteria
C3RVL1 Aphanizomenon sp./Cyanobacteria
D4TSQ4 Raphidiopsis brookii/Cyanobacteria
B3EYF9 Cylindrospermopsis raciborskii/Cyanobacteria
D6B3S9 Streptomyces albus/Actinobacteria
A3ZWL3 Blastopirellula marina/Planctomycetes
1bs0
H0IQG8 Mycobacterium abscessus/Actinobacteria
H6QZ44 Nocardia cyriacigeorgica/Actinobacteria
C1B4R7 Rhodococcus opacus/Actinobacteria
A4FHQ0 Saccharopolyspora erythraea/Actinobacteria
D9VFX1 Streptomyces sp./Actinobacteria
G8X2R2 Streptomyces cattleya/Actinobacteria
F8K2V9 Streptomyces cattleya/Actinobacteria
F8B5B8 Frankia symbiont/Actinobacteria
Q82RP2 Streptomyces avermitilis/Actinobacteria
G8SFB8 Actinoplanes sp./Actinobacteria
D6ENX9 Streptomyces lividans/Actinobacteria
O54155 Streptomyces coelicolor/Actinobacteria
B6VRQ8 Streptomyces griseoviridis/Actinobacteria
F3NN73 Streptomyces griseoaurantiacus/Actinobacteria
Q113V0 Trichodesmium erythraeum/Cyanobacteria
F4XTA7 Moorea producta/Cyanobacteria
B4WGT1 Synechococcus sp./Cyanobacteria
A3ZMA9 Blastopirellula marina/Planctomycetes
F2AMS0 Rhodopirellula baltica/Planctomycetes
Q7UYT8 Rhodopirellula baltica/Planctomycetes
D2R0Z4 Pirellula staleyi/Planctomycetes
A3ZQ92 Blastopirellula marina/Planctomycetes

Figure S2

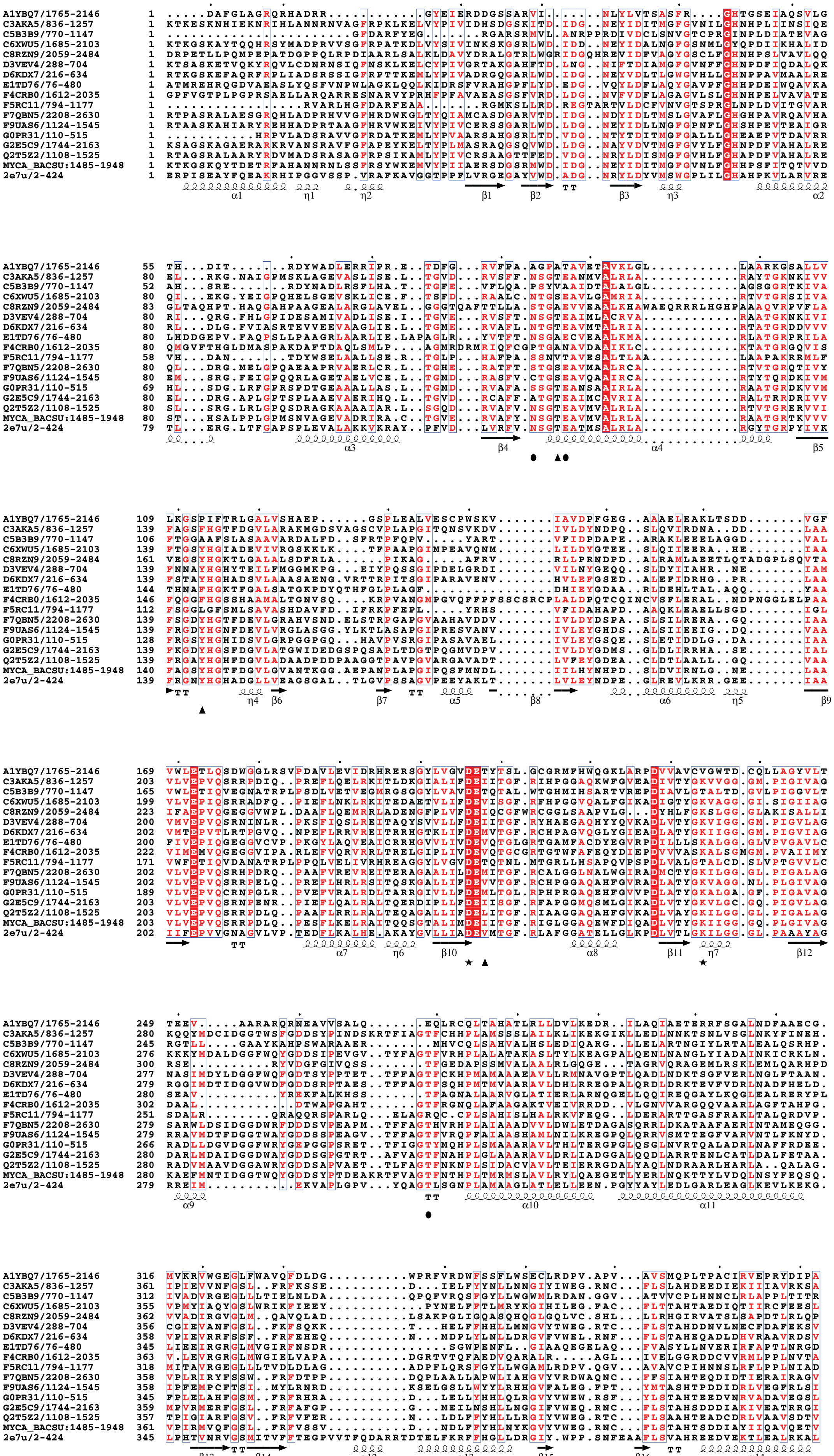

**a)**

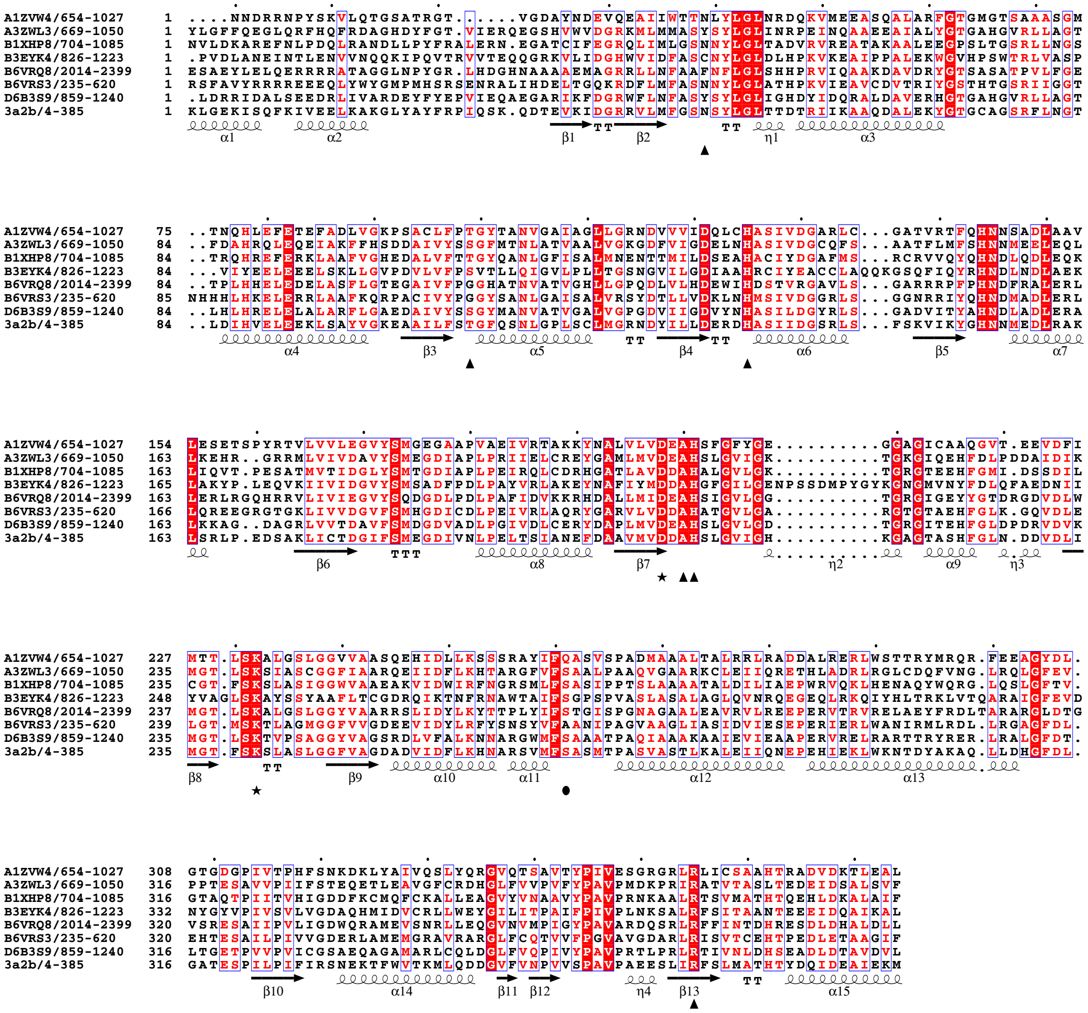
A1ZVW4/654-1027
A3ZWL3/669-1050
B1XHP8/704-1085
B3EYK4/826-1223
B6VRQ8/2014-2399
B6VRS3/235-620
D6B3S9/859-1240
3a2b/4-385

b)

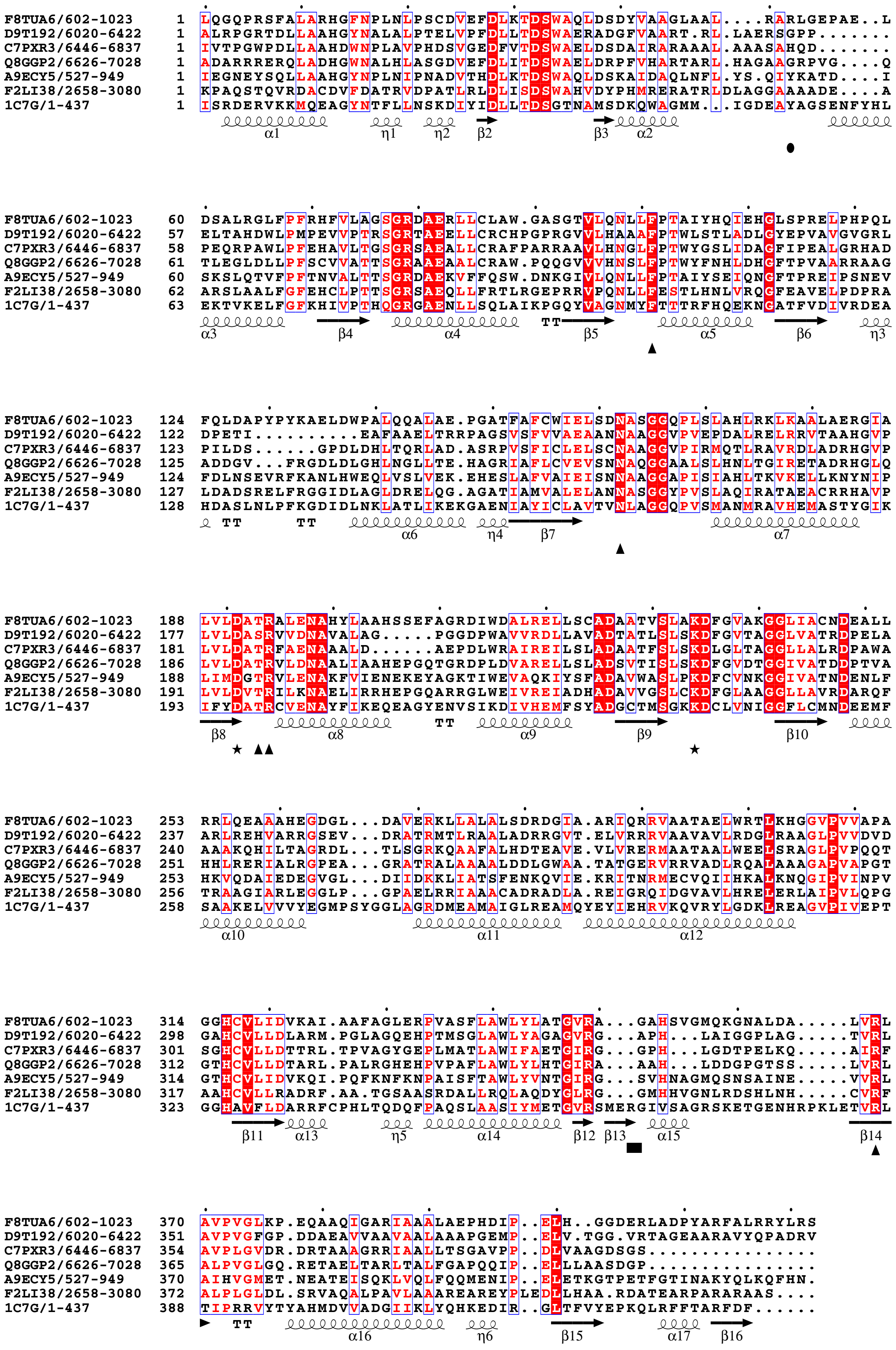
F8TUA6/602-1023 1 LQGQPRSFALARHGFNPLNLPSCDVEFDLKTDSWAQLDSDYVAAGLAAL....RARLGEPAE..L
D9T192/6020-6422 1 ALRPGRTDLLAAHGYNALALPTELVPFDLLTDSWAERADGFVAART.RL.LAERSGPP.......
C7PXR3/6446-6837 1 IVTPGWPDLLAAHDWNPLAVPHDSVGEDFVTDSWAELDSDAIRARAAAL.AAASAHQD.......
Q8GGP2/6626-7028 1 ADARRRERQLADHGWNALHLASGDVEFDLITDSWAELDRPFVHARTARL.HAGAAGRPVG.....Q
A9ECY5/527-949 1 IEGNEYSQLLAAHGYNPLNIPNADVTHDLKTDSWAQLDSKAIDAQLNFL.YS.QIYKATD....I
F2LI38/2658-3080 1 KPAQSTQVRDACDVFDATRVDPATLRLDLISDSWAHVDYPHMRERATRLDLAGGAAAADE....A
1C7G/1-437 1 ISRDERVKKMQEAGYNTFLLNSKDIYIDLLTDSGTNAMSDKQWAGMM..IGDEAYAGSENFYHL
α1 η1 η2 β2 β3 α2
F8TUA6/602-1023 60 DSALRGLFPFRHFVLAGSGRDAERLLCLAW.GASGTVLQNLLFPTAIYHQIEHGLSPRELPHPQL
D9T192/6020-6422 57 ELTAHDWLPMPEVVPTRSGRTAEELLCRCHPGPRGVVLHAAAFPTWLSTLADLGYEPVAVGVGRL
C7PXR3/6446-6837 58 PEQRPAWLPFEHAVLTGSGRSAEALLCRAFPARRAAVLHNGLFPTWYGSLIDAGFIPEALGRHAD
Q8GGP2/6626-7028 61 TLEGLDLLPFSCVVATTSGRAAEAALCRAW.PQQGVVHNSLFPTWYFNHLDHGFTPVAARRAAG
A9ECY5/527-949 60 SKSLQTVFPFTNVALTTSGRDAEKVFFQSW.DNKGIVLQNLLFPTAIYSEIQNGFTPREIPSNEV
F2LI38/2658-3080 62 ARSLAALFGFEHCLPTTSGRSAEQLLFRTLRGEPRRVPQNLLFESTLHNLVRQGFEAVELPDPRA
1C7G/1-437 63 EKTVKELFGFKHIVPTHQGRGAENLLSQLAIKPGQYVAGNMYFTTTRFHQEKNGATFVDIVRDEA
α3 β4 α4 TT β5 α5 β6 η3
F8TUA6/602-1023 124 FQLDAPYPYKAELDWPALQQALAE.PGATFAFCWIELSDNASGGQPLSLAHLRKLKAALAERGIA
D9T192/6020-6422 122 DPETI.........EAFAAELTRRPAGSVSFVVAEAANNAAGGVPVEPDALRELRRVTAAHGVP
C7PXR3/6446-6837 123 PILDS......GPDLDHLTQRLAD.ASRPVSFICLELSCNAAGGVPIRMQTLRAVRDLADRHGVP
Q8GGP2/6626-7028 125 ADDGV...FRGDLDLGHLNGLLTE.HAGRIAFLCVEVSNNAQGGAALSLHNLTGIRETADRHGLQ
A9ECY5/527-949 124 FDLNSEVRFKANLHWEQLVSLVEK.EHESLAFVAIEISNNAAGGAPISIAHLTKVKELLKNYNIP
F2LI38/2658-3080 127 LDADSRELFRGGIDLAGLDRELQG.AGATIAMVALELANNASGGYPVSLAQIRATAEACRRHAVP
1C7G/1-437 128 HDASLNLPFKGDIDLNKLATLIKEKGAENIAYICLAVTVNLAGGQPVSMANMRAVHEMASTYGIK
TT TT α6 η4 β7 α7
F8TUA6/602-1023 188 LVLDATRALENAHYLAAHSSEFAGRDIWDALRELLSCADAATVSLAKDFGVAKGGLIACNDEALL
D9T192/6020-6422 177 LVLDASRVVDNAVALAG.....PGGDPWAVVRDLLAVADTATLSLSKDFGVTAGGLVATRDPELA
C7PXR3/6446-6837 181 LVLDATRFAENAAALD......AEPDLWRAIREILSLADAATFSLSKDLGLTAGGLLALRDPAWA
Q8GGP2/6626-7028 186 LVLDATRVLDNAALIAAHEPGQTGRDPLDVARELLSLADSVTISLSKDFGVDTGGIVATDDPTVA
A9ECY5/527-949 188 LIMDGTRVLENAKFVIENEKEYAGKTIWEVAQKIYSFADAVWASLPKDFCVNKGGIVATNDENLF
F2LI38/2658-3080 191 LVLDVTRILKNAELIRRHEPGQARRGLWEIVREIADHADAVVGSLCKDFGLAAGGLLAVRDARQF
1C7G/1-437 193 IFYDATRCVENAYFIKEQEAGYENVSIKDIVHEMFSYADGCTMSGKKDCLVNIGGFLCMNDEEMF
β8 α8 TT α9 β9 β10
F8TUA6/602-1023 253 RRLQEAAAHEGDGL...DAVERKLLALALSDRDGIA.ARIQRRVAATAELWRTLKHGGVPVVAPA
D9T192/6020-6422 237 ARLREHVARRGSEV...DRATRMTLRAAALADRRGVT.ELVRRRVAAVAVLRDGLRAAGLPVVDVD
C7PXR3/6446-6837 240 AAAKQHILTAGRDL...TLSGRKQAAFALHDTEAVE.VLVRERMAATAALWEELSRAGLPVPQQT
Q8GGP2/6626-7028 251 HHLRERIALRGPEA...GRATRALAAAALDDLGWAA.TATGERVRRVADLRQALAAAGAPVAPGT
A9ECY5/527-949 253 HKVQDAIEDEGVGL...DIIDKKLIATSFENKQVIE.KRITNRMECVQIIHKALKNQGIPVINPV
F2LI38/2658-3080 256 TRAAGIARLEGGLP...GPAELRRIAAACADRADLA.REIGRQIDGVAVLHRELERLAIPVLQPG
1C7G/1-437 258 SAAKELVVVYEGMPSYGGLAGRDMEAMAIGLREAMQYEYIEHRVKQVRYLGDKLREAGVPIVEPT
α10 α11 α12
F8TUA6/602-1023 314 GGHCVLIDVKAI.AAFAGLERPVASFLAWLYLATGVRA...GAHSVGMQKGNALDA.....LVRL
D9T192/6020-6422 298 GAHCVLLDLARM.PGLAGQEHPTMSGLAWLYAGAGVRG...APH...LAIGGPLAG.....TVRL
C7PXR3/6446-6837 301 SGHCVLLDTTRL.TPVAGYGEPLMATLAWIFAETGIRG...GPH...LGDTPELKQ.....AIRF
Q8GGP2/6626-7028 312 GTHCVLLDTARL.PALRGHEHPVPAFLAWLYLHTGIRA...AAH...LDDGPGTSS.....LVRL
A9ECY5/527-949 314 GTHCVLIDVKQI.PQFKNFKNPAISFTAWLYVNTGIRG...SVHNAGMQSNSAINE.....VVRL
F2LI38/2658-3080 317 AAHCVLLRADRF.AA.TGSAASRDALLRQLAQDYGLRG...GMHHVGNLRDSHLNH.....CVRF
1C7G/1-437 323 GGHAVFLDARRFCPHLTQDQFPAQSLAASIYMETGVRSMERGIVSAGRSKETGENHRPKLETVRL
β11 α13 η5 α14 β12 β13 α15 β14
F8TUA6/602-1023 370 AVPVGLKP.EQAAQIGARIAAALAEPHDIP..ELH..GGDERLADPYARFALRRYLRS
D9T192/6020-6422 351 AVPVGFGP.DDAEAVVAAVAALAAAPGEMP..ELV.TGG.VRTAGEAARAVYQPADRV
C7PXR3/6446-6837 354 AVPLGVDR.DRTAAAGRRIAALLTSGAVPP..DLVAAGDSGS................
Q8GGP2/6626-7028 365 ALPVGLGQ.RETAELTARLTALFGAPQQIP..ELLLAASDGP................
A9ECY5/527-949 370 AIHVGMET.NEATEISQKLVQLFQQMENIP..ELETKGTPETFGTINAKYQLKQFHN.
F2LI38/2658-3080 372 ALPLGLDL.SRVAQALPAVLAAAREAREYPLEDLLHAA.RDATEARPARARAAS....
1C7G/1-437 388 TIPRRVYTYAHMDVVADGIIKLYQHKEDIR..GLTFVYEPKQLRFFTARFDF......
TT α16 η6 β15 α17 β16

c)

Figure S3

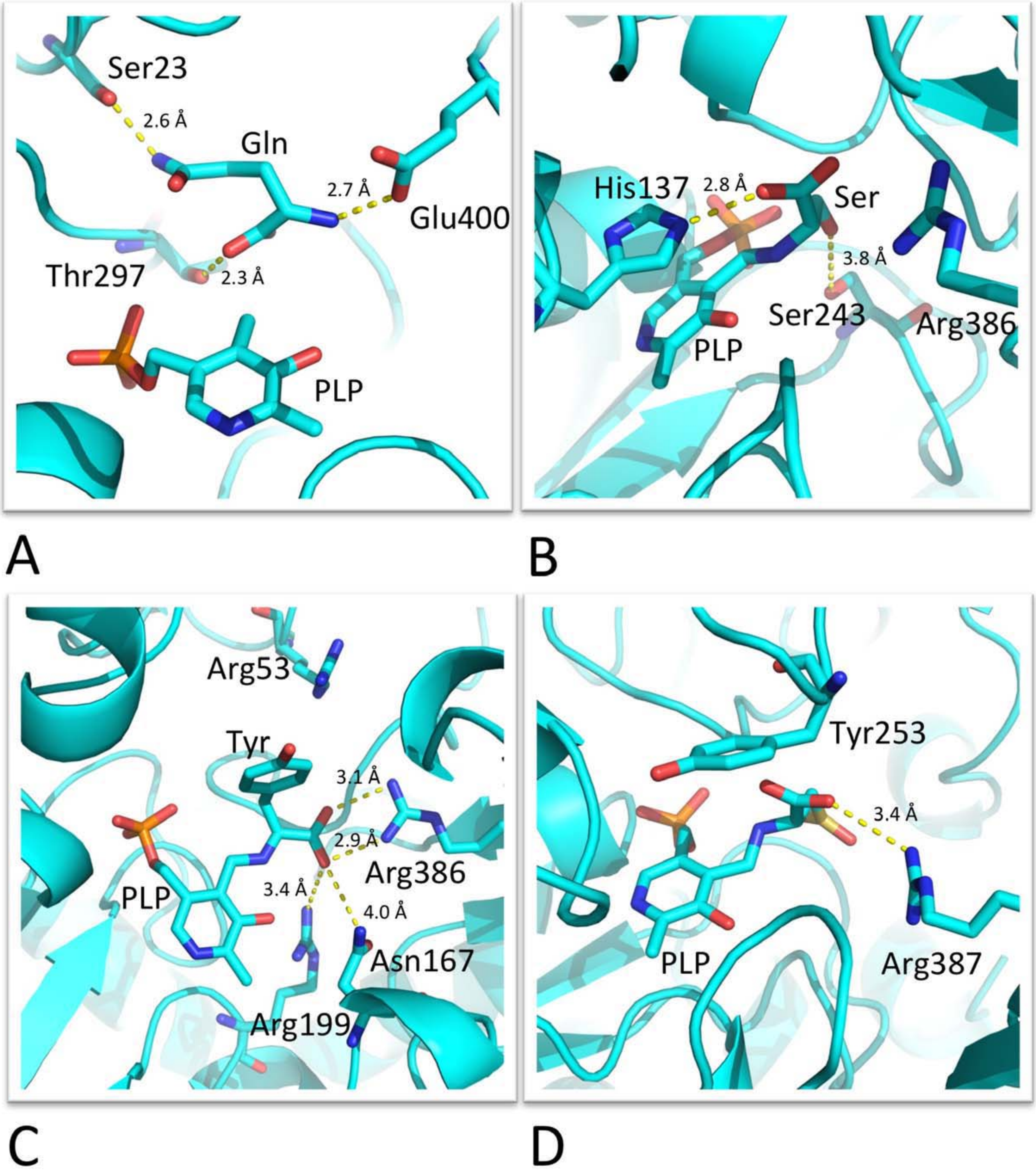
Ser23
2.6 Å
Gln
2.7 Å
Glu400
Thr297
2.3 Å
PLP
A
His137
2.8 Å
Ser
3.8 Å
Ser243
Arg386
PLP
B
Arg53
Tyr
3.1 Å
2.9 Å
Arg386
PLP
3.4 Å
4.0 Å
Asn167
Arg199
C
Tyr253
3.4 Å
PLP
Arg387
D

Figure S4

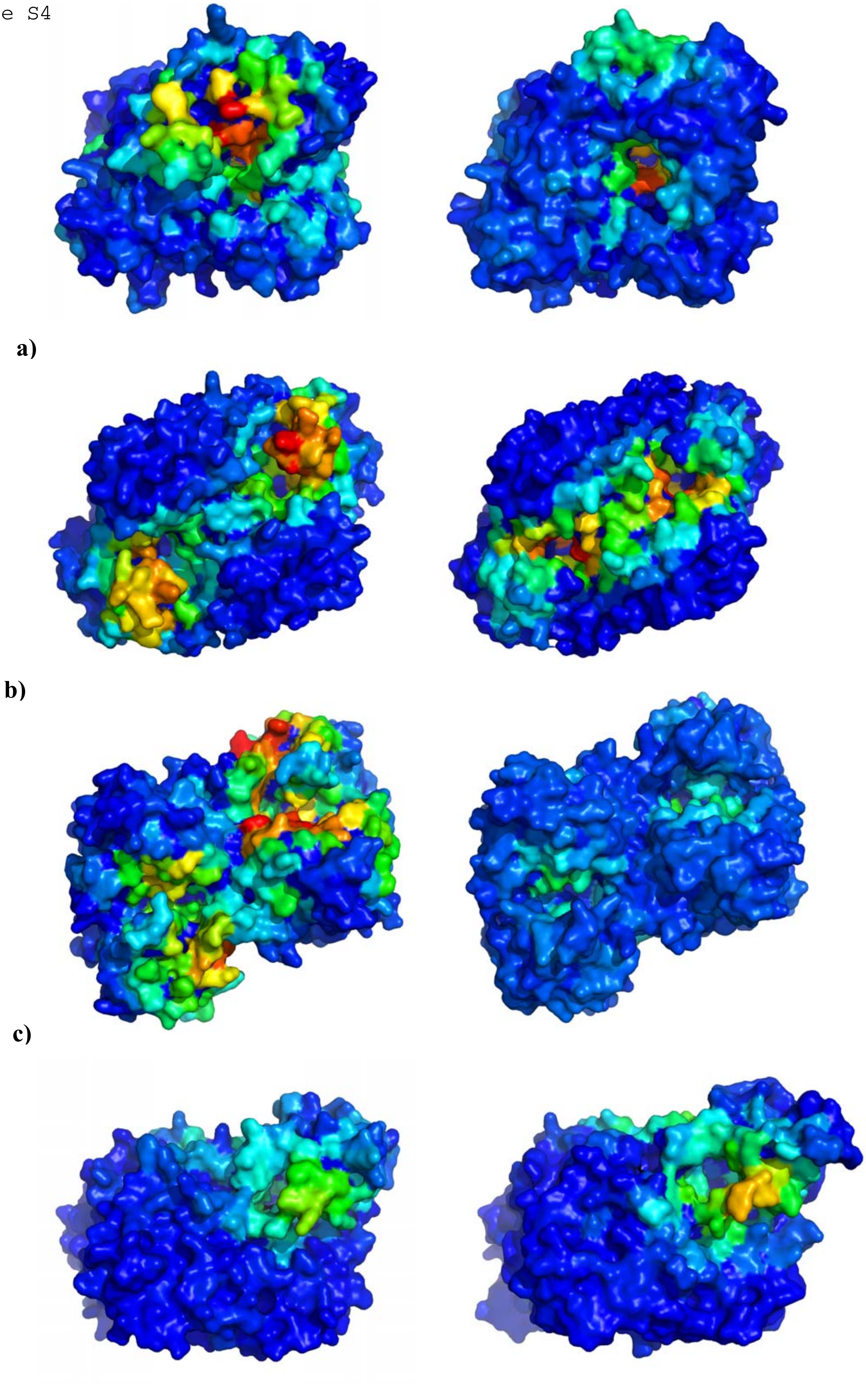

Figure S5

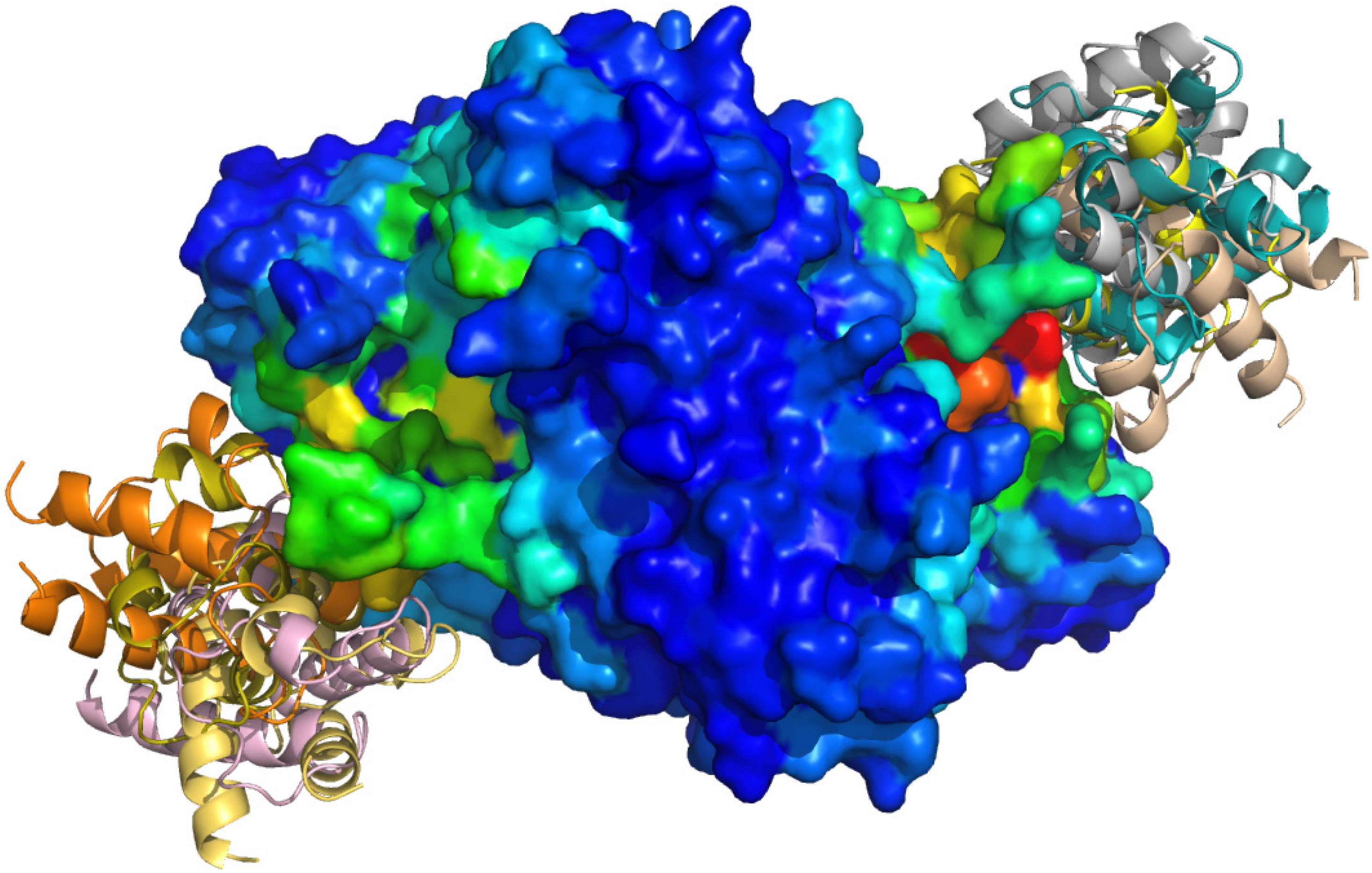

Supplement: Additional file 1: Table S1 — List of sequences of NRPS/PKS containing a type I domain. Scheme 1. Simplified scheme of typical reactions catalyzed by PLP-dependent enzymes. Figure S1. Topology of the unrooted consensus tree calculated from the multiple alignment of the entire set of type-I domains. Figure S2. Multiple alignments of the non-redundant set of sequences belonging to the three groups. Figure S3. Docking of putative substrates into the active site of the homology models of the type-I domains representative of each group. Figure S4. Prediction of the protein-protein interaction sites through the server meta-PPISP. Figure S5. Protein docking results obtained from the ClusPro server. [file 1472-6807-13-26-S1.pdf]
